# CoLoC-seq probes the global topology of organelle transcriptomes

**DOI:** 10.1093/nar/gkac1183

**Published:** 2022-12-20

**Authors:** Damien Jeandard, Anna Smirnova, Akinyemi Mandela Fasemore, Léna Coudray, Nina Entelis, Konrad U Förstner, Ivan Tarassov, Alexandre Smirnov

**Affiliations:** UMR7156 – Génétique Moléculaire, Génomique, Microbiologie (GMGM), University of Strasbourg, CNRS, Strasbourg, F-67000, France; UMR7156 – Génétique Moléculaire, Génomique, Microbiologie (GMGM), University of Strasbourg, CNRS, Strasbourg, F-67000, France; University of Würzburg, Würzburg, D-97080, Germany; UMR7156 – Génétique Moléculaire, Génomique, Microbiologie (GMGM), University of Strasbourg, CNRS, Strasbourg, F-67000, France; UMR7156 – Génétique Moléculaire, Génomique, Microbiologie (GMGM), University of Strasbourg, CNRS, Strasbourg, F-67000, France; ZB MED – Information Centre for Life Sciences, Cologne, D-50931, Germany; TH Köln – University of Applied Sciences, Faculty of Information Science and Communication Studies, Institute of Information Science, Cologne, D-50678, Germany; UMR7156 – Génétique Moléculaire, Génomique, Microbiologie (GMGM), University of Strasbourg, CNRS, Strasbourg, F-67000, France; UMR7156 – Génétique Moléculaire, Génomique, Microbiologie (GMGM), University of Strasbourg, CNRS, Strasbourg, F-67000, France; University of Strasbourg Institute for Advanced Study (USIAS), Strasbourg, F-67000, France

## Abstract

Proper RNA localisation is essential for physiological gene expression. Various kinds of genome-wide approaches permit to comprehensively profile subcellular transcriptomes. Among them, cell fractionation methods, that couple RNase treatment of isolated organelles to the sequencing of protected transcripts, remain most widely used, mainly because they do not require genetic modification of the studied system and can be easily implemented in any cells or tissues, including in non-model species. However, they suffer from numerous false-positives since incompletely digested contaminant RNAs can still be captured and erroneously identified as resident transcripts. Here we introduce Controlled Level of Contamination coupled to deep sequencing (CoLoC-seq) as a new subcellular transcriptomics approach that efficiently bypasses this caveat. CoLoC-seq leverages classical enzymatic kinetics and tracks the depletion dynamics of transcripts in a gradient of an exogenously added RNase, with or without organellar membranes. By means of straightforward mathematical modelling, CoLoC-seq infers the localisation topology of RNAs and robustly distinguishes between genuinely resident, luminal transcripts and merely abundant surface-attached contaminants. Our generic approach performed well on human mitochondria and is in principle applicable to other membrane-bounded organelles, including plastids, compartments of the vacuolar system, extracellular vesicles, and viral particles.

## INTRODUCTION

Gene expression is organized both temporally and spatially. This is especially striking in eukaryotic cells that possess several coexisting genetic systems and a developed intracellular network of membrane-bounded and membrane-less compartments. The genetic systems of the nucleus and of the genome-containing organelles (mitochondria and plastids), albeit mechanistically and functionally intertwined (e.g. via the inward protein import into the organelles, the outward retrograde signalling to the nucleus, and concerted translation programmes), remain largely segregated and tightly delimited by double membranes (1–3). Various steps of eukaryotic RNA biogenesis take place in distinct subcellular locales, including specialized nuclear condensates (such as nucleolus and Cajal bodies), cytoplasmic foci (where some snRNPs get assembled), and even the mitochondrial surface (where tRNA and piRNA maturation occurs in some species) (4–7). Mature transcripts are subject to further subcellular partitioning that brings them to their site of action, ensures their local translation, temporary sequestration, or turnover (8–10). This is achieved with the help of physical barriers (membranes, interphases), intracellular gradients of *trans*-acting factors (i.e. RNA-binding proteins, lncRNAs, ribosomes, degradation machineries), through molecular tethering of passively diffusing ribonucleoproteins (RNPs), or by their active, directed trafficking to specific subcellular locations (11,12). The importance of such an intricate organization of gene expression is highlighted by a growing number of human diseases associated with deregulations in the RNA localisation pathways (12–15). Beyond the cell limits, selective packaging of cellular RNAs into extracellular vesicles and viral particles has similarly attracted great attention from both the fundamental and applied points of view (16–26).

Understanding the general principles and the scope of RNA localisation comes by systematic, genome-wide studies. These have become possible thanks to recent advances in high-throughput methods aiming at comprehensive profiling of subcellular and extracellular transcriptomes (27,28). While some of these techniques use fluorescence *in situ* RNA hybridization to visualise selected individual transcripts (29–31), most others employ RNA-seq as a sensitive and relatively unbiased tool to identify and quantify potentially all RNAs associated with the compartment in question (32,33). The earliest and conceptually simplest method of this latter group includes a fractionation step whereby the organelle of interest (or a viral particle) is first isolated, usually treated with an RNase and/or other agents to remove co-purified, surface-attached contaminants, and analysed for its RNA content by northern blotting, RT-qPCR or deep sequencing (21–25,34–42). More recently, proximity labelling techniques, such as APEX-RIP, APEX-Seq, Proximity-CLIP, Cap-seq and proximity-specific ribosome profiling, enabled high-resolution *in situ* subcellular RNA profiling based on the deposition of covalent tags on RNPs found in immediate vicinity of a genetically engineered enzyme targeted to the organelle of interest. These powerful methods have drawn first nearly comprehensive RNA landscapes of mitochondria, endoplasmic reticulum, nucleus, cytosol and even distinct subnuclear territories (43–50). In many cases, they also provided access to the localisation topology of organelle-associated transcripts by distinguishing internal RNAs, present in the luminal space of organelles, and external, surface-attached RNAs. This property is biologically very important as it determines which transcripts and proteins can in principle interact in the cellular milieu, eventually producing physiologically relevant outcomes, and which should never come in touch with each other.

Each of the above approaches has its strengths and weaknesses (33,51). Fractionation-based techniques perform well on most membrane-bounded organelles and even on some viruses with robust, RNase-proof proteinaceous capsids (52–54). They produce high amounts of analysable material and do not require any genetic modification of the studied system, which facilitates their implementation in genetically intractable organisms and tissues. On the other hand, with the advent of RNA-seq they increasingly fall prey to the high sensitivity of this technique, which can detect even low-level contaminating RNA species and falsely declare them as resident transcripts. By contrast, proximity approaches identify organellar RNAs with exquisite spatial precision and even perform reasonably well on membrane-less compartments which do not tolerate RNase treatment (46,55). However, proximity labelling techniques require genetic introduction of the tagging enzyme and its specific, infallible localisation to the compartment of interest. This restricts their use to those model species for which appropriate genetic engineering tools and good knowledge of protein localisation pathways are available (so-far they have been used essentially in cultured human and yeast cells). Furthermore, biochemical tagging is usually a low-yield, random process, and the existing proximity approaches typically focus on long polyadenylated transcripts, which biases them against shorter, noncoding, or less abundant RNA species (45,49,50). Therefore, although the introduction of these methods was truly transformative for the field of subcellular localisation, more comprehensive, unbiased, and widely applicable subcellular transcriptomic tools are in demand to complement the existing techniques and extend their scope to non-model organisms and less tractable compartments.

**Figure 1.**
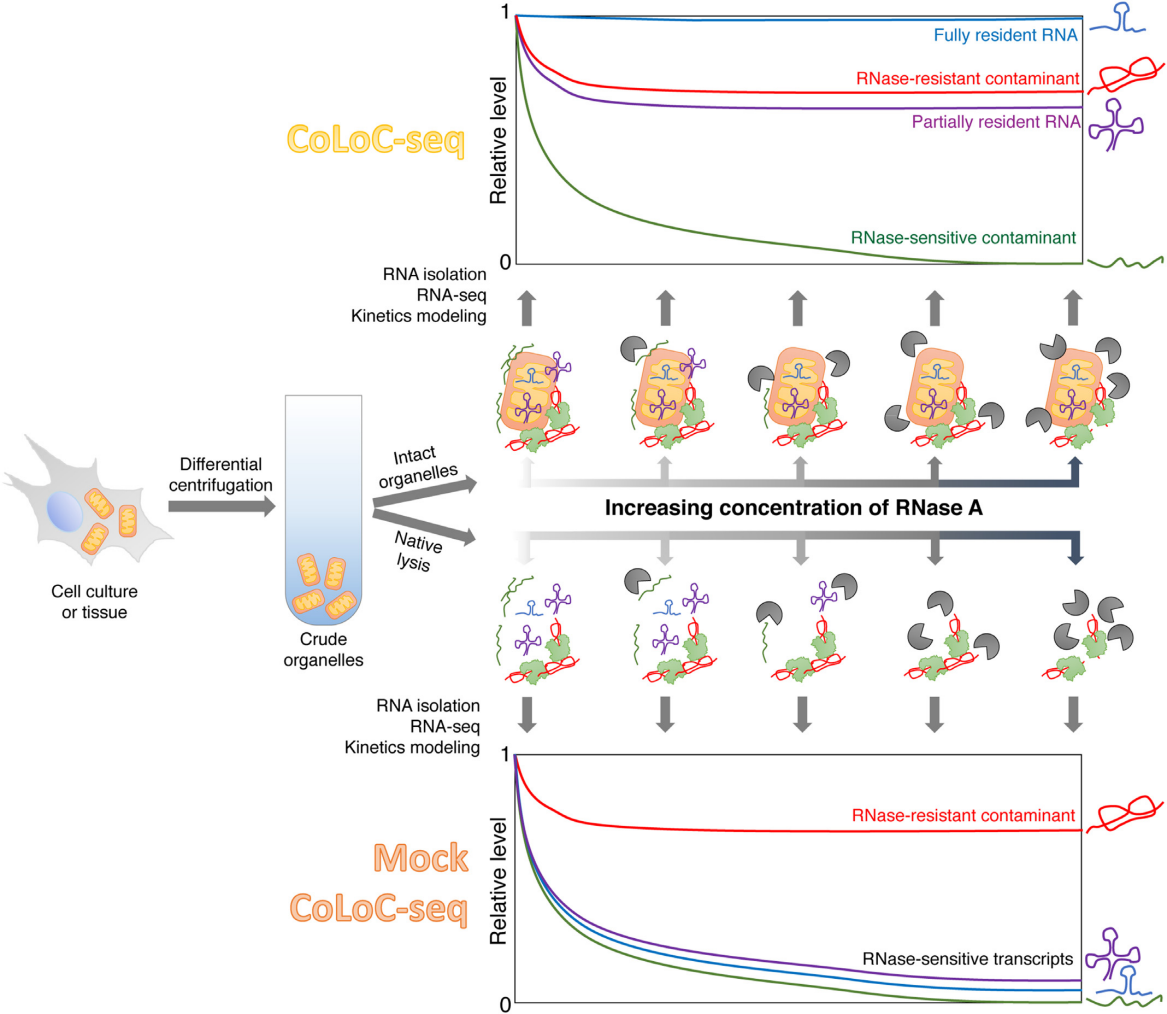
CoLoC-seq pipeline. The topology of a subcellular transcriptome, associated with an organelle of interest, can be interrogated by performing two types of controlled, kinetically resolved RNase treatment reactions. To this end, crude organelles (e.g. mitochondria) are isolated from a cell culture or a tissue and divided in two portions to follow either the CoLoC-seq or the Mock CoLoC-seq protocol. In the CoLoC-seq setup (*top*), a preparation of intact organelles is split in a series of identical samples subjected, during a given time, to a gradient of RNase A concentrations (including an untreated sample). This creates characteristic digestion dynamics which, for each RNA species, depends on its exact location and its accessibility to the RNase. These dynamics can be analysed transcriptome-wide by deep sequencing of remaining intact RNA in each sample, followed by standard kinetics mathematical modelling to infer the reaction rate and the protection status of individual RNA species (see Materials and Methods for model details). Resident transcripts (*blue*), shielded by the organellar membranes, do not participate in the reaction and remain at a constant level. By contrast, typical RNase-sensitive surface-exposed contaminants (*green*) follow a gradual, pseudo-first order decay. Partially resident transcripts (*violet*) combine the two behaviours by producing an intermediate-level plateau. In the Mock CoLoC-seq protocol (*bottom*), the preparation of organelles is first mildly solubilised to destroy membranes and only then subjected to a similar gradient of RNase A concentrations as in CoLoC-seq. In this case, all transcripts should in principle be accessible to the RNase and take part in the reaction, yielding a pseudo-first order decay curve. However, if an RNA species is intrinsically RNase-resistant (e.g. due to its tight structure or its protection by proteins), it will plateau in both CoLoC-seq and Mock CoLoC-seq experiments, independently of the membrane integrity (*red*).

Here, we describe an alternative organelle transcriptomics approach that preserves the key advantages of fractionation-based techniques while alleviating their main shortcoming – poor control over rampant false-positives. This generic method, called Controlled Level of Contamination coupled to deep sequencing (CoLoC-seq), instead of aspiring to fully decontaminate purified organelles (which is practically unfeasible), leverages the basic enzymatic kinetics of RNase digestion and infers the localisation topology of transcripts from their biochemical reactivity across a gradient of RNase concentrations (Figure 1). Exposing organelle-associated transcripts to an added RNase creates unique, RNA-specific depletion dynamics, which depends on the size of the RNA pool available to degradation and on the cleavage rate constant. The ensemble of these degradation kinetics is analysed transcriptome-wide with a dedicated RNA-seq pipeline, and a simple kinetics model is then fitted to measure the reactivity and the localisation topology of each RNA. Under this setup, contaminant RNAs follow gradual, pseudo-first order depletion dynamics, which betrays them even when they cannot be completely digested within the allotted time. By contrast, resident transcripts show a plateau equivalent to the pool of molecules protected from the RNase by the organellar membranes. Thus, regardless of the absolute numbers of reads mapping to each transcript from the RNase-treated organelles, one can distinguish between *bona fide* resident (luminal) RNAs and merely abundant surface-attached contaminants. We applied this method to human mitochondria and successfully detected all long and the majority of short mitochondrial transcripts as genuinely resident, validating our strategy. Moreover, CoLoC-seq confidently classified all long and most smaller nuclear-encoded RNA species associated with mitochondria as contaminants (even when they remained numerically abundant in the ‘purified’ organelles), while providing evidence for partial mitochondrial localisation of a small subset of RNA polymerase III-generated transcripts, including Y RNAs and select tRNAs. The generic nature of CoLoC-seq makes it in principle applicable to other membrane-bounded organelles, extracellular vesicles, and virions, independently of their origin and prior knowledge.

## MATERIALS AND METHODS

### Reagents

#### Commercial enzymes and other recombinant proteins used in this study

DNase I (Thermo Fisher Scientific, Waltham, USA, Cat # EN0525), Micrococcal nuclease (Thermo Fisher Scientific, Waltham, USA, Cat # 88216), RNA 5’-pyrophosphohydrolase (New England Biolabs, Ipswich, USA, Cat # M0356S), RNase A (Thermo Fisher Scientific, Waltham, USA, Cat # EN0531), RNase A/T_1_ mix (2 mg/ml, 5,000 U/ml; Thermo Fisher Scientific, Waltham, USA, Cat # EN0551), RNase One (Promega, Madison, USA, Cat# M4261), RNase T_1_ (Thermo Fisher Scientific, Waltham, USA, Cat # EN0541), SUPERase·In RNase Inhibitor (Thermo Fisher Scientific, Waltham, USA, Cat # AM2694).

#### Kits and other reagents used in this study

AMPure XP kit (Beckman Coulter, Indianapolis, USA, A63881), Bradford assay ROTI Nanoquant (Carl Roth, Karlsruhe, Germany, Cat # K880.1), Lipofectamine 2000 (Thermo Fisher Scientific, Waltham, USA, Cat # 11668019), ProLong Gold Antifade Mountant (Thermo Fisher Scientific, Waltham, USA, Cat # P36930), Protein A-Sepharose 6MB (Sigma-Aldrich, Saint Louis, USA, Cat # P6649), Roti Aqua-P/C/I (Carl Roth, Karlsruhe, Germany, Cat # X985.1), SuperSignal West Pico Chemiluminescent Substrate (Thermo Fisher Scientific, Waltham, USA, Cat # 34579), TRIzol reagent (Thermo Fisher Scientific, Waltham, USA, Cat # 15596026), ViewRNA ISH Cell assay kit (Thermo Fisher Scientific, Waltham, USA, Cat # QVC0001).

#### Antibodies used in this study

mouse monoclonal α-CYCS (BD Pharmingen, Franklin Lakes, USA, Cat # 556432), goat polyclonal α-ENO (Santa Cruz Biotechnology, Inc, Santa Cruz, USA, Cat # sc-7455), mouse monoclonal α-His (Sigma-Aldrich, Saint Louis, USA, Cat # SAB1305538), mouse monoclonal α-LRPPRC (Santa Cruz Biotechnology, Inc, Santa Cruz, USA, Cat # sc-166178), rabbit polyclonal α-OPA1 (Proteintech Group, Rosemont, USA, Cat # 27733-1-AP), rabbit polyclonal α-PNPase (Abcam, Cambridge, UK, Cat # ab96176), mouse monoclonal α-Ro60 (Santa Cruz Biotechnology, Inc, Santa Cruz, USA, Cat # sc-100844), rabbit polyclonal α-Ro60 (Sigma-Aldrich, Saint Louis, USA, Cat # HPA002835), rabbit polyclonal α-TOMM20 (Santa Cruz Biotechnology, Inc, Santa Cruz, USA, Cat # sc-11415), rabbit polyclonal α-uL4m (Proteintech Group, Rosemont, USA, Cat # 27484-1-AP), polyclonal sheep HRP-conjugated α-mouse (GE Healthcare, Chicago, USA, Cat # NXA931), polyclonal donkey HRP-conjugated α-rabbit (GE Healthcare, Chicago, USA, Cat # NA934V).

#### Specialised commercial instruments used in this study

ChemiDoc Touch Imaging System (Bio-Rad, Hercules, USA, Cat # 1708370), LSM 780 microscope (Carl Zeiss, Oberkochen, Germany), NextSeq 500 (Illumina, San Diego, USA), Optima XPN-100 ultracentrifuge (Beckman Coulter, Brea, USA), Typhoon Trio Variable Mode Imager System (GE Healthcare, Chicago, USA).

### Biological resources

#### Cell lines used in this study

Flp-In T-REx 293 cell line (Thermo Fisher Scientific, Waltham, USA, Cat # R78007), SAL004 (Flp-In T-REx 293-derived PDHA1-mCherry-expressing; see section ‘Creation of a PDHA1-mCherry-expressing cell line’).

#### Plasmids used in this study

mCherry-PDHA1-N-10 (Addgene, Cambridge, USA, Cat # 55118), pcDNA5 FRT/TO (Thermo Fisher Scientific, Waltham, USA, Cat # V652020), pOG44 (Thermo Fisher Scientific, Waltham, USA, Cat # V600520), ASP0042 (derived from mCherry-PDHA1-N-10 and pcDNA5 FRT/TO).

### Cell culturing

Human Flp-In T-REx 293 cells, derived from the HEK293 cell line, were cultivated at 37°C in the presence of 5% CO_2_ in Eagle's Minimum Essential Medium (EMEM, Sigma) containing 1 g/l glucose and supplemented with 1.5 g/l sodium bicarbonate and 0.11 g/l sodium pyruvate. One complete set of CoLoC-seq and Mock CoLoC-seq samples requires ∼2500 cm² of nearly confluent cells (equivalent of ∼3 × 10^8^ HEK293 cells). All cultures were routinely verified by PCR for mycoplasma contamination, as described (56). At 80% confluence, the cells were harvested by incubation with 2.5 mM EDTA in PBS for 20 min at 37°C, centrifuged at 600 g for 10 min at room temperature, and washed once with PBS.

HepG2 and SAL004 cells for microscopy experiments were cultured in standard Dulbecco's modified Eagle's medium (DMEM) containing 4.5 g/l glucose, supplemented with 10% fetal bovine serum, Penicillin-Streptomycin (Sigma-Aldrich) and Amphotericin B (Sigma-Aldrich), at 37°C, 5% CO_2_.

### Creation of a stable PDHA1-mCherry-expressing cell line

A gene for pyruvate dehydrogenase E1 subunit alpha 1 fused N-terminally to mCherry (PDHA1-mCherry) from the plasmid mCherry-PDHA1-N-10 was amplified with primers Anja0062 and Anja0063 (Supplementary Table S1) and recloned into pcDNA5 FRT/TO. The resulting plasmid is referred to as ASP0042. For creation of a stable cell line, Flp-In T-REx 293 cells were transfected with a mixture of 0.4 μg of ASP0042 and 3.6 μg of pOG44 (encoding Flp-recombinase) with 10 μl of Lipofectamine 2000 in 2 ml of OptiMEM (Gibco) in 9 cm^2^ Petri dishes. After 6 h of transfection, the medium was changed to EMEM and the cells were left to propagate for 48 h. Then the cells were trypsinized, diluted, and reseeded in the presence of 320 μg/ml hygromycin B Gold (InvivoGen). In 48 h, concentration of hygromycin was reduced to 160 μg/ml, and 18 days after transfection, a population of clones was isolated and propagated in the presence of 160 μg/ml hygromycin. The resulting cell line used in this study is referred to as SAL004. PDHA1-mCherry expression was induced with 0.2 mg/ml tetracycline for 24 h.

### Preparation of crude mitochondria

Crude mitochondria were isolated by differential centrifugation, following established protocols (57–60), with modifications. All procedures were carried out at 4°C, unless specified differently. Harvested cells were resuspended in 30 ml of ice-cold Buffer A (10 mM Tris–HCl, pH 6.7, 0.6 M sorbitol) and disrupted at high speed with a pre-chilled laboratory blender (Waring Commercial) three times for 15 s (with 1–2 min intervals to avoid overheating). Cellular debris and nuclei were removed by three 3-min centrifugations at 1000 g, the supernatant being every time transferred into a fresh tube. The mitochondria were then pelleted by centrifugation at 21 000 g for 30 min. The crude mitochondrial pellet was carefully but thoroughly resuspended in 8 ml of Buffer A and split in four 2-ml portions. Each portion was loaded on a two-cushion sucrose gradient formed of 10 ml of Buffer B (10 mM Tris–HCl, pH 6.7, 1.65 M sucrose) and 15 ml of Buffer C (10 mM Tris–HCl, pH 6.7, 0.6 M sucrose) in SW 32 Ti tubes (Beckman Coulter). Upon 1-h centrifugation at 45 000 g, the mitochondria-containing interphase was collected. The interphases were washed with excess of Buffer A and centrifuged at 21 000 g for 30 min. The supernatant was discarded.

### CoLoC procedure

The mitochondrial pellets were combined and gently but thoroughly resuspended in 800 μl of Buffer A. 500 μl of the suspension were saved for a regular CoLoC-seq experiment, whereas the remaining 300 μl were reserved for the accompanying mock CoLoC-seq experiment. The concentration of the first portion was adjusted with Buffer A to ∼1.6 mg/ml of mitochondrial protein (as measured by Bradford assay). The resulting suspension was split in a series of identical 80-μl samples (normally, ∼10 samples must be sufficient to create an informative depletion curve). In parallel, a series of 80-μl RNase A dilutions in Buffer D (10 mM Tris–HCl, pH 6.7, 0.6 M sorbitol, 200 mM NaCl, 2 mM EDTA) were prepared with concentrations ranging from 0 to 6 μg/ml. For the experiments described in this paper, the following concentrations have been applied: CoLoC-seq #1 – 0, 0.2, 0.6, 1.2, 2.0, 2.6, 3.2, 4.0 and 6.0 μg/ml; CoLoC-seq #2 – 0, 0.1, 0.2, 0.6, 1.2, 2.0, 2.6, 3.2, 4.0 and 6.0 μg/ml. The mitochondrial suspensions and the RNase A dilutions (pre-warmed to 25°C for 1 min) were mixed 1:1 and incubated for 10 min on a water bath at 25°C. The reactions were then diluted with 1.6 ml of pre-chilled Buffer E (10 mM Tris–HCl, pH 6.7, 0.6 M sorbitol, 5 mM EDTA) and centrifuged at 16 000 g for 20 min. The pellet was thoroughly resuspended in 100 μl of Buffer F (10 mM Tris–HCl, pH 6.7, 0.6 M sorbitol, 1 mM EDTA), and RNA was extracted with TRIzol (Invitrogen), following the manufacturer's instructions. Each extracted RNA sample was mixed with the identical amount (150 ng for CoLoC-seq #1, 90 ng for all other experiments) of a yeast tRNA-sgRNA chimeric spike-in transcript (5′-GAGAAGUAAGCACUGUAAAGGUUUUAGAGCUAGAAAUAGCAAGUUAAAAUAAGGCUAGUCCGUUAUCAACUUGAAAAAGUGGCACCGAGUCGGUGCUUGCCUUGUUGGCGCAAUCGGUAGCGCGUAUGACUCUUAAUCAUAAGGUUAGGGGUUCGAGCCCCCUACAGGGCUCCA), which does not cross-map to the human genome (61), to enable data normalization. The samples were treated with 1 U of DNase I in the presence of 20 U of SUPERase·In, re-extracted with TRIzol, and stored at –80°C.

### Technical note

There are two important considerations to take into account when choosing the RNase for CoLoC-seq experiments: (i) it should have properties of a kinetically perfect enzyme to enable straightforward data analysis (see the section ‘Kinetics model derivation and fitting’) and (ii) it should generate 5’-hydroxyl and 2’/3’-phosphate termini to permit selective sequencing of intact transcripts (see the section ‘Library preparation and RNA-seq’). Other RNases creating 5’-hydroxyl and 2’/3’-phosphate ends (micrococcal nuclease, RNase I, RNase T_1_) had been tested as well but were found to be overall less active and more idiosyncratic than RNase A (Supplementary Figure S1), in agreement with previous reports (62,63). The choice of RNase concentrations depends on the specific activity of the enzyme batch and the nature of the material to treat and should be adjusted individually for every new application. It must enable the observation of a gradual digestion dynamics of contaminant transcripts without compromising the quality of the final RNA samples.

### Mock CoLoC procedure

For the mock CoLoC-seq experiment, the 300 μl of crude mitochondrial suspension were mixed with 300 μl of ice-cold Buffer H (10 mM Tris–HCl, pH 6.7, 0.6 M sorbitol, 1% *n*-dodecyl-β-d-maltoside) and lysed on ice with a small Dounce homogenizer (50 strokes). The lysate was cleared by two 20-min centrifugation at 16 000 g, at 4°C. The cleared lysate was adjusted to ∼1.6 mg/ml of protein (as measured by Bradford assay) with Buffer A and split in a series if identical 80-μl samples. The RNase A treatment was performed in the same way as in the standard CoLoC procedure. The following RNase A concentrations (before mixing with the mitochondrial lysate) have been used for the mock CoLoC-seq #1 – 0, 0.2, 0.6, 1.2, 2.0, 2.6, 4.0, 4.6, 5.2 and 6.0 μg/ml; for the mock CoLoC-seq #2 – 0, 0.1, 0.2, 0.6, 1.2, 2.0, 2.6, 3.2, 4.0 and 6.0 μg/ml. After the 10-min incubation at 25°C, the reactions were centrifuged for 40 min at 16 000 g at 4°C (to subject them to conditions similar to the regular CoLoC procedure). RNA was then extracted with TRIzol and stored, as described above.

### Northern blotting

Northern blotting was performed as described (64). For this, ∼1 μg of the untreated (‘0’) RNA samples were mixed 1:1 with 0.025% SDS, 18 mM EDTA, 0.025% bromophenol blue in deionised formamide, denatured for 5 min at 95°C, and loaded onto a 6% gel in 1× TBE with 8 M urea. All the other, RNase-treated, samples were loaded in the same volume fraction to enable their direct comparison. Upon migration and ethidium bromide staining, RNA was transferred onto a nylon Amersham HybondN+ membrane (GE Healthcare) and UV-crosslinked. Pre-hybridization was carried out in 6× SSC containing 5× Denhardt's solution and 0.2% SDS at 65°C for 30 min. To probe for specific transcripts, the membrane was incubated overnight with continuous rotation with a 5’-[^32^P]-labelled oligonucleotide probe (see Supplementary Table S1) in 3× SSC, 0.1% SDS, 0.5× TE, 0.5 M NaCl, 5× Denhardt's solution at 42°C. After hybridization, the membrane was washed with 5× SSC, 0.1% SDS, dried, and exposed with a Phosphorimager plate. The radioactive signal was visualised on Typhoon Trio (GE Healthcare) and analysed with ImageQuant TL (v. 7.0, GE Healthcare). The spike-in RNA signal was used for normalization across the entire sample range and the reconstruction of depletion dynamics for each transcript. To re-probe for a different RNA, membranes were stripped in stripping buffer (1% SDS, 0.1× SSC, 40 mM Tris–HCl, pH 7.6) at 80°C three times for 10 min and washed once in 2× SSC.

### Library preparation and RNA-seq

cDNA library preparation and RNA-seq were performed by Vertis Biotechnologie AG (Freising, Germany). Supplementary Figure S2 shows the key library preparation steps that ensure the selective sequencing of intact transcripts. Briefly, cap structures were first removed with RNA 5’-pyrophosphohydrolase. The 5’-adapter was ligated to 5’-phosphorylated ends. Then the 3’-adapter was ligated to 3’-hydroxyl ends. The first-strand cDNA synthesis was performed with M-MLV reverse transcriptase with a 3’-adapter-annealing primer. The resulting cDNA was PCR-amplified (15 cycles) with a high-fidelity DNA polymerase and barcoded TruSeq primers (Supplementary Table S1). cDNA was purified with the AMPure XP kit, fragmented, end-repaired, and subjected to another round of adapter ligation and PCR amplification. The cDNA samples were pooled equimolarly and size-selected on an agarose gel in the range 10-to-220 nt (excluding the flanking sequences). The pool was sequenced on an Illumina NextSeq 500 instrument (75-nt single-end reads).

Although the fragmentation step had been expected to destroy the strand-specificity of the protocol, in reality, mapping results showed the sequencing reads to cluster on the 5’-end of the annotated genes in sense orientation (Supplementary Figures S3 and S4). Therefore, the initial 5’-adapter ligation largely determined the strandedness of the reads, which permitted to exclude eventual ambiguity in transcript assignment and quantification.

### CoLoC-seq data processing

Sequencing read libraries were pre-processed with cutadapt version 2.8 (65) to trim adapter sequences. Thereafter, read alignment and gene feature quantification was done with READemption version 0.4.3 (66) (https://doi.org/10.5281/zenodo.250598). All libraries were aligned to the Human genome (Genome Reference Consortium Human Build 38 patch release 13) retrieved from RefSeq (67). READemption uses segemehl version 0.2.0–418 (68) as the read aligner. The options used for alignment, coverage calculation, and feature quantification can be found in the scripts deposited at Zenodo (https://doi.org/10.5281/zenodo.6389451).

Entries for repetitive genes were compounded, and their reads were summed up. Genes for which the majority of reads came from cross-mapping to embedded tRNA-, snRNA-, 7SL-, 5S rRNA- or mtDNA-like (NUMTs) sequences were excluded from the analysis. A cut-off of 30 reads was applied to all untreated samples to ensure robust starting level measurement (smaller cut-offs resulted in the inclusion of lower-quality, noisy profiles, and were, therefore, abandoned). Read counts in each library were normalised by the corresponding number of reads mapping to the spike-in RNA to reconstruct digestion profiles. The RNA level in the untreated samples (0 μg/ml RNase A) was set to 1. Read distributions across select genomic loci were visualised in the Integrated Genome Browser (v. 9.1.8).

### Kinetics model derivation and fitting

Classical degradation of an *i*th transcript by a kinetically perfect enzyme (whose catalysis rate is only limited by substrate binding and not by the chemical reaction itself), such as RNase A (69), is described by the standard equation:

$$\begin{equation*}{\rm{\ }}\frac{{d{R}_i}}{{dt}} = \ - {k}_i{R}_iA\end{equation*}$$

where *R_i_* is the concentration of the *i*th RNA, *A* is the RNase A concentration (assumed to remain constant during digestion), and *k_i_* is the corresponding pseudo-first order rate constant.

Separation of variables and integration by time results in the canonical exponential decay function:

$$\begin{equation*}{\rm{\ }}{R}_t = {R}_0\ {e}^{ - {k}_iAt}\end{equation*}$$

where *R_0_* and *R_t_* are the concentrations of the *i*th RNA at the time points 0 and *t*.

The ratio $f( A )\ = \ \frac{{{R}_t}}{{{R}_0}}$ is experimentally measurable and represents the proportion of intact *i*th RNA remaining after digestion with the *A*th concentration of RNase A at the time point *t*. However, if the reaction system contains a pool of *i*th transcript of the size *r*, which does not take part in the reaction (e.g. protected by organellar membranes), this ratio will assume a slightly different form:

$$\begin{eqnarray*}f\ \left( A \right) & = & \ \ \frac{{{R}_t + r}}{{{R}_0 + r}} = \frac{{{R}_0{e}^{ - {k}_iAt} + r}}{{{R}_0 + r}}\ = \frac{{{R}_0}}{{{R}_0 + r}}\ {e}^{ - {k}_iAt}\nonumber\\ && + \ \frac{r}{{{R}_0 + r}} = \left( {1 - {P}_0} \right)\ {e}^{ - {k}_iAt} + {P}_0\end{eqnarray*}$$

where the constant ${P}_0 = \ \frac{r}{{{R}_0 + r}}$ corresponds to the initial proportion of the *i*th RNA that is in principle unavailable for digestion by RNase.

If one fixes time (e.g. to *t* = 10 min, as in this study), the model will have only two parameters to be determined, based on experimentally measured remaining proportion *f*(*A*) versus RNase A concentration *A*:

(1) $$\begin{equation*}f\ \left( A \right) = \left( {1 - {P}_0} \right)\ {e}^{ - k{^{\prime}}_iA} + {P}_0\end{equation*}$$

where *k’*_*i*_ reflects the rate with which RNase A digests the *i*th transcript, and *P*_0_ measures the relative size of the protected pool, which is the parameter of interest of CoLoC-seq.

Remark that when an RNA is not localised inside the organelle, i.e. its *P*_0_ = 0, the equation resolves back into the canonical pseudo-first-order decay:

(2) $$\begin{equation*}f\ \left( A \right) = {e}^{ - k{^{\prime}}_iA}\ \end{equation*}$$

By contrast, transcripts fully localised inside the organelle and inaccessible for RNase A (*P*_0_ = 1) are expected to show a constant level independent of RNase A concentration:

(3) $$\begin{equation*}f\ \left( A \right) = {P}_0\ = \ 1\end{equation*}$$

Nonlinear curve fitting of Model 1 into CoLoC-seq and Mock-CoLoC-seq data (for either individual or combined replicates) was performed in Origin 2021b (v9.8.5.212, OriginLab Corporation) with the following settings. Constraints on parameters: 0 ≤ *P*_0_ ≤ 1, *k′* ≥ 0; Leverberg Marquardt regression method; initial *P_0_* estimate fixed to the lowest observed *f*(*A*) value; 500 iterations; tolerance 1E-09; ‘Model comparison method’ for CI computation for the parameters. In a few cases where the fit did not converge or the dependency between parameters exceeded 0.3 (which precludes their reliable determination and suggests that the model is unnecessarily complex), the simpler Model 2 was fitted without constraints. High and significant *P*_0_ values (Model 1) or small and/or insignificant *k′* values (both models) indicate that the transcript level is largely stable and insensitive to RNase A digestion. Note that the Model 3 is implicitly tested as alternative (*R*² = 0) in both Model 1 and Model 2.

### Submitochondrial fractionation for RNA and protein localisation

The submitochondrial fractionation was performed as in (64,70), with modifications to assess the RNA localisation. Flp-In T-REx 293 cells (225 cm²) were grown to 80–90% confluency, harvested, resuspended in 1.5 mL of ice-cold Buffer F (0.6 M sorbitol, 10 mM HEPES–KOH, pH 7.5, 1 mM EDTA), and disrupted on ice with a 2-ml syringe and a 26G × 25 mm needle by 20 strokes. The disrupted cells were centrifuged at 600 g for 10 min at 4°C, then at 1000 g for another 10 min to sediment nuclei and cell debris. The pre-cleared supernatant was then centrifuged at 14 000g for 20 min at 4°C to sediment mitochondria. The mitochondrial pellet was thoroughly resuspended in 500 μl of Buffer F, and its protein content was measured by Bradford assay. The mitochondrial suspension was then split in three aliquots of 167 μl, diluted with 1 ml of Buffer F to each new tube and centrifuged at 14 000 g for 10 min. The pellets were resuspended at ∼2 mg of mitochondrial protein per ml in either of the three buffers: sample #1 in the isotonic Buffer F, sample # 2 in the hypotonic Buffer G (10 mM HEPES–KOH, pH 7.5, 1 mM EDTA), sample #3 in the lysis Buffer H (10 mM HEPES–KOH, pH 7.5, 1 mM EDTA, 0.5% *n*-dodecyl-β-maltoside). The latter sample was thoroughly lysed with a 2-ml syringe and a 26G × 25 mm needle by 10 strokes to ensure complete membrane solubilisation. Each of these three samples was then split in three identical aliquots, of which one was left untreated, one was added 1 μl of RNase A/T_1_ mix (2 mg/ml RNase A, 5000 U/ml RNase T_1_; Thermo Fisher Scientific), and one was added 5 μl of RNase A/T_1_ mix. All aliquots were incubated at 25°C for 10 min. Then 1.5 ml of TRIzol reagent was added, and RNA was extracted following the manufacturer's instructions. All RNA samples were dissolved in 35 μl of RNase-free water, and 10 μl of each sample was resolved in denaturing 8 M urea 6% PAGE followed by northern blotting, as described above.

To assess the submitochondrial localisation of proteins, the three mitochondrial samples resuspended in Buffers F, G, H were each split in two aliquots. One of the aliquots was left untreated, whereas the other was added 50 μg/ml proteinase K. Both aliquots were incubated on ice for 20 min. Then 1 mM PMSF and ¼ volume of 100% trichloroacetic acid (prepared by dissolving 5 g of dry TCA in 3.5 ml of water) were added to all the samples, mixed well, and incubated for another 10 min on ice to stop digestion and precipitate proteins. The digests were centrifuged for 10 min at 14 000 g at 4°C. The pellets were washed twice with ice-cold acetone, dried overnight on the bench, and resuspended in 100 μl of 1× Laemmli buffer. For analysis, the samples were incubated at 80°C for 5–10 min, briefly sonicated to disperse the pellets, and 10 μl of each sample was resolved by 10% (for large proteins) or 15% (for small proteins) SDS-PAGE, followed by semi-dry transfer onto an Amersham Protran blotting nitrocellulose membrane (GE Healthcare). The membrane was blocked with 1 × TBS containing 0.05% Tween-20 and 10% skimmed milk and incubated with antibodies against Ro60 and established markers of mitochondrial subcompartments in 1× TBS, 0.05% Tween-20 for 1 h at room temperature. After a wash with 1× TBS, 0.05% Tween-20 for 30 min, secondary HRP-conjugated antibodies were applied to the membrane in the same way, and the resulting chemiluminescent signals were visualised with the help of SuperSignal West Pico Chemiluminescent Substrate (Thermo Fisher Scientific) on ChemiDoc Touch Imaging System (Bio-rad), further analysed in Image Lab (v. 5.2.1).

### Coimmunoprecipitation of Ro60 complexes

Coimmunoprecipitation was performed as described in (64). Crude mitochondria were isolated from 450 cm² of Flp-In T-REx 293 cells grown to ∼80% of confluence, as described in the section ‘Submitochondrial fractionation for RNA and protein localisation’. The mitochondria were completely lysed by resuspension in 1 ml of Buffer I (20 mM Tris–HCl, pH7.5, 150 mM KCl, 1 mM MgCl_2_, 1 mM DTT, 0.5% *n*-dodecyl-β-d-maltoside, 1 mM PMSF) and disruption with a small Dounce homogeniser (20 strokes). The lysate was cleared by centrifugation at 14 000 g for 10 min at 4°C. A 10-μl sample was collected and mixed with 90 μl of 1× Laemmli buffer. For RNA analysis, a 100-μl sample was mixed with 1 ml of TRIzol to extract RNA. The lysate was pre-treated by incubation with 75 μl (bed volume) of protein A-sepharose beads (Sigma-Aldrich, pre-washed 5 times with 1 ml of Buffer J: 20 mM Tris–HCl, pH7.5, 150 mM KCl, 1 mM MgCl_2_, 1 mM DTT, 1 mM PMSF) at 4°C for 30 min to remove unspecifically interacting proteins. The pre-treated lysate was then split in two equal portions: one portion was added 30 μl of a control antibody (goat polyclonal α-ENO, Santa Cruz Biotechnology, Inc, Cat # sc-7455) and the other with 30 μl of mouse monoclonal α-Ro60 antibody (Santa Cruz Biotechnology, Inc., Cat # sc-100844). The samples were rocked for 30 min at 4°C, then each mixed with 75 μl (bed volume) of protein A-sepharose beads (pre-washed 5 times with 1 ml of Buffer J) and rocked for another 30 min at 4°C. The beads were pelleted by centrifugation at 14 000 g for 30 s. A 10-μl and a 100-μl samples were collected and treated as previously for subsequent protein and RNA analyses. The beads were washed with 5 × 1 ml of Buffer J, and the last wash samples were collected and treated in the same way. The beads were resuspended in 0.5 ml of Buffer J, added 0.5 ml of phenol:chloroform:isoamyl alcohol (P:C:I, 25:24:1, Carl Roth), shaken vigorously for 20 s, and left on the bench for 3 min to initiate phase separation. The samples were centrifuged at 15 200g for 30 min at 15°C. The aqueous phases were collected, added 50 μg/ml of glycogen, and RNA was precipitated with 750 μl of isopropanol overnight at –20°C. Precipitated RNA was pelleted by centrifugation at 15 200 g for 20 min at 4°C, washed with 80% and 100% ethanol, dried, and solubilised in 35 μl of RNase-free water. The organic phase and the interphase after the P:C:I extraction were thoroughly mixed with 1.5 ml of ice-cold acetone, and the proteins were precipitated overnight at -20°C. Precipitated proteins were pelleted by centrifugation at 15 200 g for 30 min at 4°C, washed twice with ice-cold acetone, dried on the bench for 8 h and solubilised in 150 μl of 1× Laemmli buffer. For RNA analysis, 5 μl of each RNA sample were resolved by a denaturing 6% PAGE followed by northern blotting, as described above. For protein analysis, all samples were boiled at 80°C for 5 min, and 20 μl of lysate, flow-through and wash samples were resolved along with 40 μl of coIP samples by 10% SDS-PAGE followed by western blotting, as described in the section ‘Submitochondrial fractionation for RNA and protein localisation’.

Coimmunoprecipitation of Ro60 complexes from total cell lysates for validation of the mouse α-Ro60 antibody (Santa Cruz Biotechnology, Inc, Cat # sc-100844) was performed similarly, with the same amount of starting material and an α-His antibody (Sigma-Aldrich, Cat # SAB1305538), which does not have antigens in the Flp-In T-REx 293 cell line, as negative control. Coimmunoprecipitates were analysed by LC–MS/MS as described in (64). The spectral counts for each protein detected at FDR <1% in the α-Ro60 and α-His samples were normalised by the total number of spectral counts and compared. Proteins enriched >2-fold in the α-Ro60 sample, with non-overlapping 95% Poissonian confidence intervals, were deemed significantly enriched.

### Immunofluorescence, single-molecule RNA FISH and microscopy

Immunofluorescence and smFISH were performed in HepG2 cells as described in (64,71). Briefly, cells were seeded on an 8-well Nunc Lab-Tek slide (Thermo Fisher Scientific) 24-to-48 h prior the experiment, then fixed by incubation with a 3% formaldehyde solution diluted with the DMEM medium (4% paraformaldehyde dissolved in PBS by heating at 60°C and adjusted to 3% with DMEM) for 12 min at 37°C. For immunostaining, the cells were permeabilised for 10 min at room temperature with 0.3% Triton X-100 solution in 1× PBS. After blocking at room temperature for 30 min with 5% bovine serum albumin (BSA) in 1× PBS, the samples were incubated for 1-to-3 h with primary antibodies diluted in the blocking buffer. They were then incubated with the corresponding secondary antibody conjugated with Alexa Fluor 488, 555 or 647. Each step alternated with five washes with 1× PBS. Branched DNA RNA smFISH combined with antibody-mediated protein detection (71,72) was performed with the ViewRNA ISH Cell assay kit (Thermo Fisher Scientific), following the manufacturer's protocol (see Supplementary Table S1 for further information about the probe sets used).

For mitoplast isolation, induced SAL004 cells, cultivated in a 75 cm² flask, were detached with ice-cold PBS-EDTA, centrifuged and resuspended in 1 ml of MitoBuffer (0.6 M sorbitol, 1 mM EDTA, 10 mM Tris–HCl, pH 7.5) supplemented with 0.3% BSA. Cells were disrupted on ice with a 2-ml syringe and a 26G × 25 mm needle by 20 strokes. Cell debris and nuclei were removed by two 5-min centrifugations at 1500 g at 4°C. The supernatant was centrifuged for 5 min at 13 000 g at 4°C to pellet mitochondria. The crude mitochondrial pellet was resuspended 300 μl of MitoBuffer, and an equal volume of the 2× RNase A solution (10 μg/ml RNase A in Mitobuffer supplemented with 4 mM MgCl_2_) was added. After incubation for 7 min at room temperature, 800 μl of MitoBuffer supplemented with 2.5 mM EDTA was added, and mitochondria were again pelleted at 13 000 g for 5 min at 4°C. Then mitochondria were washed three times with 500 μl of ice-cold MitoBuffer, resuspended in MitoBuffer containing 0.00625% digitonin and incubated for 8 min at room temperature to permeate the outer membrane. The resulting mitoplasts were diluted with 1 ml of ice-cold MitoBuffer, collected by centrifugation at 13 000 g for 5 min at 4°C, resuspended in 100 μl of MitoBuffer and immediately transferred to 8-well LabTek or 10-well Grenier slides, pre-treated with poly-l-lysine. Mitoplasts were fixed for 30 min and then probed for specific RNAs with custom Alexa Fluor 647-conjugated branched DNA oligo sets (Supplementary Table S1) using a ViewRNA ISH Cell assay kit as above but without protein labelling.

Samples were imaged on an LSM 780 microscope (Carl Zeiss) under a 63×/1.4 oil objective in ProLong Gold Antifade Mountant (Invitrogen). The fluorescent labels were excited at 635 nm (Alexa Fluor 647), 561 nm (mCherry) or 488 nm (Alexa Fluor 488), and their emission was collected (650–750 nm, 570– 630 nm, 500–540 nm) with PMT detectors. Image segmentation and quantification of subcellular shapes was done by Squassh (73) in a plugin MosaicSuit in Fiji (74). For smFISH analysis of mitoplast-associated RNAs, 7-to-10 frames with a total of 739-to-1477 mitoplasts were quantified.

### Statistical analyses

Violin plots were created in Microsoft Power BI Desktop (v. 2.71.5523.821) with the use of the Epanechnikov kernel density method. Spearman's correlation was calculated with Free Statistics and Forecasting Software, v1.2.1 (http://www.wessa.net/rwasp_spearman.wasp). Mann–Whitney test was performed in Statistics Kingdom (https://www.statskingdom.com/170median_mann_whitney.html). One-sample Wilcoxon test and the calculation of confidence intervals (95% CI) for colocalised proportions of transcripts detected by smFISH were performed in GraphPad Prism 9 (v9.3.1). The *P*_0_*–k* simulation in Supplementary Figure S7B was performed in MS Excel 2016 based on the Model 1 and visualised with Heatmapper (http://heatmapper.ca/). Ensemble RNA folding free energy was predicted with RNAfold (75). RNA sequences were retrieved from RefSeq (67).

## RESULTS

### General principle of CoLoC-seq

In an idealised fractionation experiment, organelles are isolated and treated with an RNase to degrade contaminant RNAs remaining on their surface, leaving resident transcripts, protected by the organellar membranes, intact. Those surviving RNAs can then be purified and analysed, for example, by northern blotting. In reality, due to differences in sequence, structure and involvement in RNPs, various cellular RNAs have unequal sensitivity to RNase treatment, some being digested slowly, if at all. Such transcripts present a challenging confounding factor for fractionation-based subcellular transcriptomics since they may be erroneously identified as resident, especially when more sensitive amplification-based detection methods, such as RT-qPCR and RNA-seq, are employed.

To control for such false-positives and rigorously assess the complement of *bona fide* organelle-resident RNAs, CoLoC-seq introduces several key innovations in the above experimental design. The CoLoC-seq pipeline (Figure 1, top) starts canonically with the isolation of crude organelles, e.g. by differential centrifugation or affinity purification. However, instead of relying on a single snapshot trying to catch a unique RNase treatment condition where all contaminants would be fully digested (which is practically unattainable), CoLoC-seq creates a series of reaction snapshots to directly measure the biochemical reactivity of each RNA toward the RNase using classical enzymatic kinetics and thereby estimate the size of the RNA pool that does not take part in the reaction (i.e. remains unavailable to the RNase). To this end, the initial organelle prep is split in a series of identical samples and treated with increasing concentrations of an RNase (including an untreated sample as reference). After a defined time, the reactions are stopped, RNA is isolated and quantitatively analysed by northern blotting or RNA-seq. By assessing the dynamics with which intact transcripts disappear upon the increase of the RNase concentration, one can, by fitting a simple kinetics model (see Model 1 in Materials and Methods), estimate for each detected RNA two parameters: its digestion rate constant (*k’*), which reflects its sensitivity to RNase, and the proportion of the RNA that is protected from digestion (*P*_0_), which is our parameter of interest. When *P*_0_ = 0, this means that the entire RNA pool participates in the reaction: one observes a gradual disappearance of the transcript in a classical pseudo-first-order decay, indicating that it is a contaminant (Model 2 in Materials and Methods). By contrast, when *P*_0_ >> 0, this indicates that there exists a sizable pool of protected RNA: the curve will plateau at a non-null level, suggesting that this transcript is genuinely present inside the organelle (with *P*_0_ = 1 corresponding to 100% of RNA protected from digestion; Model 3 in Materials and Methods). Thereby, looking at the detailed depletion dynamics of each RNA, one can tell genuinely resident transcripts from even slowly digested contaminants (which may still remain numerically abundant at the highest RNase concentration).

To verify that the protection observed by CoLoC-seq results from the organellar membranes and not from other intrinsic factors (e.g. an intricate tertiary structure or shielding by associated proteins), a Mock CoLoC-seq experiment is performed in parallel (Figure 1, bottom). In this case, a portion of the initial organelle prep is mildly lysed with a detergent under native conditions to dissolve the membranes while minimally perturbing molecular interactions. After this, the RNase treatment and all the subsequent steps are performed in the same way as in a normal CoLoC-seq experiment. In the Mock CoLoC-seq scenario, all transcripts become accessible to the RNase (*P*_0_ = 0) and are expected to show gradual, pseudo-first-order digestion as the RNase concentration increases. If, however, a plateau is still observed (*P*_0_ >> 0), this means that the corresponding RNA is intrinsically resistant to the RNase and may, therefore, represent a false-positive finding. By these means, CoLoC-seq efficiently controls for the two most important sources of false-positive identifications in fractionation-based transcriptomic approaches – slowly digested and RNase-resistant transcripts.

### CoLoC-seq analysis in human mitochondria: proof of principle

We set out to evaluate the feasibility of CoLoC-seq on a relatively well-studied human mitochondrial transcriptome. Mitochondria possess their own genome, providing a straightforward way of benchmarking for *bona fide* resident transcripts. Indeed, human mitochondria (especially from the HEK293 cell line used here) are a popular test organelle for subcellular transcriptomics techniques (45–47,49), which permits us to directly compare the performance of CoLoC-seq with that of other existing tools. Of no less interest, a number of reports provided evidence for the presence of select nuclear DNA (nDNA)-encoded transcripts inside these organelles, although this topic remains contentious and, therefore, deserves more rigorous assessment (51,76).

**Figure 2.**
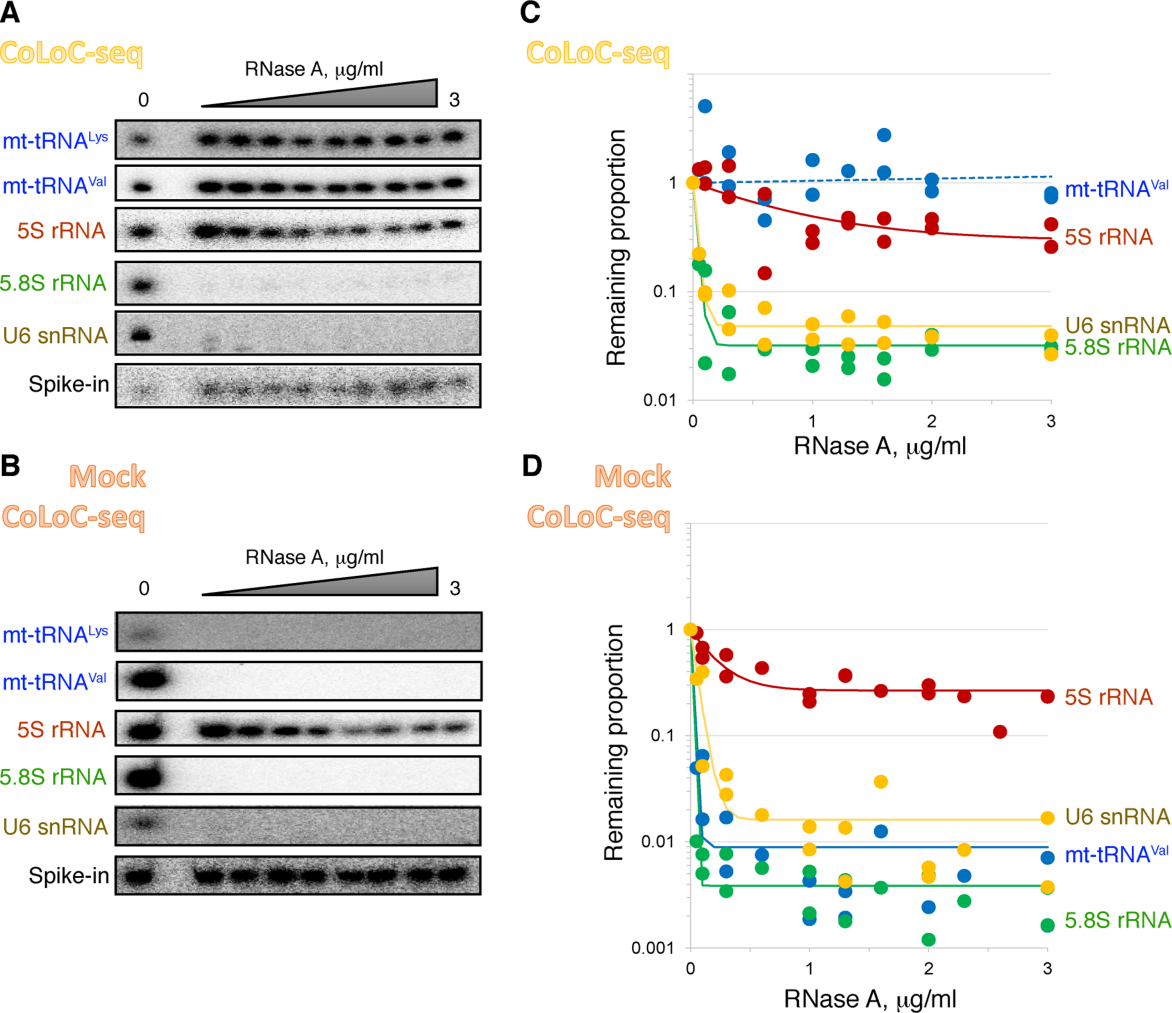
CoLoC-seq faithfully recapitulates the depletion dynamics of topologically different mitochondria-associated RNAs. (**A, B**) Northern blot analysis of a representative CoLoC-seq (A) and the corresponding Mock CoLoC-seq (B) experiments showing how different RNAs react to RNase A in the presence (A) or in the absence (B) of intact mitochondrial membranes. mtDNA-encoded transcripts are highlighted in blue, and nDNA-encoded RNAs are shown in other colours. (**C, D**) Corresponding CoLoC-seq and Mock CoLoC-seq profiles showing the RNA levels measured by deep sequencing in two compounded replicates (*n* = 19 for each transcript). Full and broken lines show the fitted kinetics Models 1 and 2, respectively (see also Supplementary Table S2 for values and parameters).

We isolated crude mitochondria from HEK293 cells using a classical protocol, including differential sedimentation and an isopycnic centrifugation step in a sucrose gradient (see Materials and Methods for details). A portion of this mitochondrial prep was lysed to perform a Mock CoLoC-seq experiment. Both lysed and intact mitochondria were divided in 9–10 identical samples and treated with increasing concentrations (from 0 to 3 μg/ml) of RNase A. In 10 min the reactions were stopped, RNA was isolated, spiked-in with a T7 transcript without homology to the human genome (combining parts of a CRISPR guide RNA and a yeast tRNA), and analysed by northern blotting and RNAs-seq (Figure 2). As expected, the mitochondrial DNA (mtDNA)-encoded mt-tRNA^Val^ and mt-tRNA^Lys^ were fully resistant to RNase A in the CoLoC-seq setup (Figure 2A) but rapidly degraded in Mock CoLoC-seq (Figure 2B), indicating that they are genuinely present inside the organelles. By contrast, abundant nDNA-encoded 5.8S rRNA and U6 snRNA were rapidly degraded in both cases (Figure 2A, B), confirming that they are surface-attached degradable contaminants. Of note, 5S rRNA, which had been proposed to partially localise in mammalian mitochondria (36,57,77–82), plateaued at an intermediate level in both the CoLoC-seq and the Mock CoLoC-seq experiments (Figures 2A, B). This indicates that it remained to a large extent resistant to RNase degradation, even when the mitochondrial membranes had been solubilised, and may, therefore, represent an example of a false-positive identification (see also Supplementary Figure S1). These results show that the CoLoC-seq procedure can qualitatively distinguish between the three main kinds of degradation dynamics produced by topologically different RNA species, as predicted from kinetics principles (Figure 1 and Model 1).

We then subjected the same samples to Illumina sequencing. Since the quantity of interest is the amount of remaining intact RNA, we developed a custom protocol that excludes RNase A-cleaved transcripts (Supplementary Figure S2 and Materials and Methods). To this end, we leveraged the specific chemistry of RNase A cleavage: the enzyme generates 5’-hydroxyl and 3’-phosphate groups, which are incompatible with standard adaptor ligation (63). Thereby only 5’-phosphorylated and 3’-hydroxylated RNAs get amplified, sequenced, and quantified. Importantly, a single RNase A cleavage is sufficient to invalidate the transcript and exclude it from the library.

Upon mapping the reads to the human genome and their cross-library normalisation with the help of an exogenously added spike-in RNA, we could reconstitute the genome-wide digestion dynamics of mitochondria-associated transcripts (Supplementary Table S2). The obtained profiles faithfully recapitulated the behaviour of the select RNA species measured by northern blotting: U6 snRNA and 5.8S rRNA were rapidly degraded to background levels in both CoLoC-seq and Mock CoLoC-seq experiments; 5S rRNA resisted full degradation in both cases; mt-tRNA^Val^ remained insensitive to RNase A treatment in CoLoC-seq but quickly disappeared in Mock CoLoC-seq (Figure 2). The overall agreement in RNA proportion measurements between the conventional technique (northern blotting) and CoLoC-seq was very good (Spearman's *ρ* = 0.888, *P* = 8.7 × 10^−73^), with 90.6% of proportion measurements being within 0.3 from each other (Supplementary Figure S5A). As a more stringent test, we fitted the kinetics model into the data quantified by northern blotting or by CoLoC-seq and found that the resulting *P*_0_ values were in general highly concordant (Spearman's *ρ* = 0.822, *P* = 1.6 × 10^−5^) and did not show signs of significant bias (Supplementary Figure S5B). These results indicate that CoLoC-seq correctly and quantitatively captures the kinetic behaviour of analysed transcripts, evaluates the size of the protected RNA pool, and thereby distinguishes between resident RNAs, degradable and non-degradable contaminants.

### CoLoC-seq analysis of the entire human mitochondrial transcriptome

**Figure 3.**
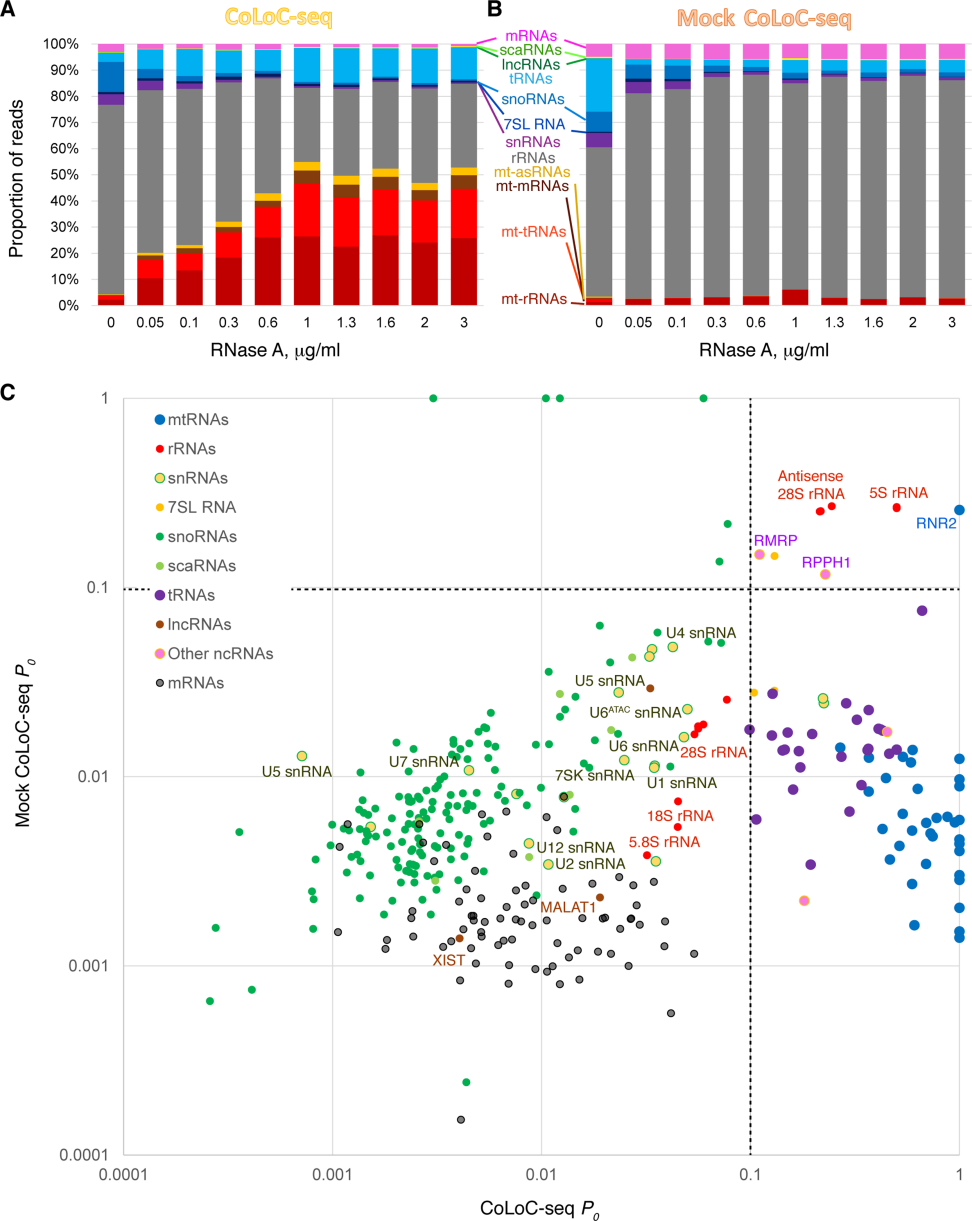
RNA localisation topology landscape of mitochondria-associated transcripts. (**A, B**) The evolution of library composition across the gradient of RNase A concentrations in CoLoC-seq (A) and Mock CoLoC-seq (B). Only in CoLoC-seq, mtDNA-encoded transcripts get progressively enriched, while most nDNA-encoded RNA classes are depleted. (**C**) 350 mitochondria-associated transcripts are plotted according to their protected proportions *P*_0_ measured in CoLoC-seq and Mock CoLoC-seq. The probability of mitochondrial residency increases from left to right; the intrinsic resistance of transcripts to RNase A increases from bottom to top. See also Supplementary Table S2 for the underlying data and Figure 4A for a more detailed view of the lower right quadrant, where candidate resident RNAs are located.

We extended our CoLoC-seq pipeline to interrogate the genome-wide topology of mitochondria-associated RNAs relative to the mitochondrial membranes. We expectedly observed that in CoLoC-seq libraries the proportion of mtDNA-derived reads increases as the RNase A concentration grows. This corresponds to the selective protection of mitochondria-localised transcripts against the backdrop of the gradual bulk degradation of surface-exposed RNAs (Figures 3A). This enrichment, however, did not occur in Mock CoLoC-seq libraries, indicating a similar susceptibility of mtDNA- and nDNA-encoded transcripts to degradation once the mitochondrial membranes are gone (Figure 3B).

Using a stringent read count cut-off to enable robust profiling and model fitting (see Materials and Methods), we identified 351 non-redundant transcripts as significantly associated with mitochondria, including 35 mtDNA-encoded ones, which corresponds to 13,338 individual RNA level measurements (Supplementary Table S2). The CoLoC-seq replicates were well-correlated with each other (Spearman's *ρ* = 0.774 ± 0.036, mean ± SD for RNase A concentration-matched samples); so were the Mock CoLoC-seq replicates (Spearman's *ρ* = 0.700 ± 0.112, Supplementary Figure S6A). By contrast, the CoLoC-seq samples were markedly different from the Mock CoLoC-seq ones (Spearman's *ρ* = 0.383 ± 0.104; two-tailed Mann-Whitney test *P* = 1.7 × 10^−6^), indicating globally dissimilar digestion patterns (Supplementary Figure S6A). Furthermore, within each CoLoC-seq or Mock CoLoC-seq dataset, samples treated with different RNase A concentrations also tended to correlate with each other, whereas there was little cross-correlation between CoLoC-seq and Mock CoLoC-seq samples (two-tailed Mann-Whitney test *P* = 4.4 × 10^–16^, Supplementary Figure S6A). This suggests that the ensemble of interrogated RNAs followed consistent reaction paths along the entire range of RNase A concentrations, and these dynamics differed between CoLoC-seq and Mock CoLoC-seq, as could already be inferred from library compositions (Figure 3A, B).

We then automatically fitted the CoLoC-seq kinetics models into these data to estimate the key parameters of the corresponding reaction curves: the protected proportion *P*_0_ and the effective digestion rate constant *k’* (see Materials and Methods). The biological replicates returned highly concordant *P_0_* values (Spearman's *ρ* = 0.815 ± 0.042, mean ± range), whereas CoLoC-seq and Mock CoLoC-seq samples were more dissimilar (Spearman's *ρ* = 0.47 ± 0.14, Supplementary Figure S6B). This remaining correlation is not surprising, given that the majority of mitochondria-associated RNAs behave as contaminants and follow similar digestion dynamics with *P_0_* values close to 0 in both CoLoC-seq and Mock CoLoC-seq setups (see below).

Due to the high similarity of replicates, we could merge them and thereby increase statistical power and the accuracy of kinetics estimates. Unsupervised fitting of the complete Model 1 was successful in 679 cases (96.7% out of 351 CoLoC-seq and 351 Mock CoLoC-seq profiles). An additional 22 profiles, for which *P*_0_ and *k’* were too strongly intertwined (suggesting that in their case the Model 1 was unnecessarily complex), could be rescued with the simpler Model 2 (see Materials and Methods), bringing the overall fitting success rate to 99.9%. The latter was in particular the case for many mtDNA-encoded RNAs in CoLoC-seq experiments, where their apparent digestion rate approached 0 (due to protection by mitochondrial membranes) and the profile remained largely flat (*R²* = –0.12 ± 0.22, mean ± SD, Supplementary Table S2).

We wondered whether the digestion rate constants we obtained by fitting the kinetics models were biochemically meaningful. The vast majority of rate constants measured in our CoLoC-seq and Mock CoLoC-seq experiments were fairly close to *k*_cat_*/K*_m_ reported for a standard RNase A substrate, UpA (2.3 × 10^6^ M^−1^ s^−1^, Supplementary Figure S7A) (83). Only in rare cases, e.g. for 5S rRNA (*k* = 1.9 × 10^4^ M^−1^ s^−1^), was the apparent digestion rate worse than that of a poor RNase A substrate, UpOC_6_H_4_-*p*-NO_2_ (*k*_cat_*/K*_m_ = 5.7 × 10^4^ M^−1^ s^−1^) (83), confirming its remarkable recalcitrance to digestion (Figure 2). We then tested whether the observed rate constants permitted independent estimation of the size of protected RNA pools *P*_0_. By simulating data for a continuum of *k–P*_0_ combinations, we observed that the CoLoC-seq Model 1 remains robust at *k* > 10^5^ M^−1^ s^−1^ but becomes uncertain when digestion occurs too slowly (Supplementary Figure S7B). Nearly all experimental *k* values measured by CoLoC-seq and Mock CoLoC-seq fell within the optimal dynamic range, enabling unequivocal, untangled *P*_0_ estimation. We found that *P_0_* estimates were not appreciably influenced by RNA abundance, pyrimidine content, or overall amount of structure (Supplementary Figure S7C–E). Altogether, these results indicate that CoLoC-seq is a reproducible approach, capturing biochemically meaningful parameters from a myriad of simultaneously going RNase digestions reactions, that can be used for a reliable and unbiased estimation of accessible and inaccessible RNA pools.

To globally visualise the localisation topology of mitochondria-associated transcripts we plotted the protected RNA pools *P*_0_, as measured in CoLoC-seq *versus* Mock CoLoC-seq (Figure 3C). Thereby we could roughly partition all transcripts in three unequal groups. The majority of RNAs (∼77%), had low *P*_0_ values (<0.1) in both CoLoC-seq and Mock CoLoC-seq experiments, which identifies them as trivial degradable contaminants. This group includes all long nDNA-encoded transcripts (mRNAs, lncRNAs), 5.8S rRNA, 18S rRNA, 28S rRNA, snRNAs, snoRNAs and scaRNAs. The absence of long nDNA-encoded RNAs inside human mitochondria has also been observed by proximity labelling approaches, corroborating our observation (45–47,49). Another, small, group of RNAs on the opposite extreme of the diagonal showed relatively high *P_0_* values in both CoLoC-seq and Mock CoLoC-seq, meaning that they are at least partially intrinsically resistant to RNase A, independently of the integrity of the mitochondrial membranes (Figure 3C). Strikingly, this group contains three small noncoding RNAs (ncRNAs), which are most frequently cited as potentially imported into the mammalian mitochondria: 5S rRNA, RMRP (RNA component of RNase MRP) and RPPH1 (RNA component of the nuclear RNase P) (36,51,57,77–82,84–86). Their localisation in this quadrant suggests that they may in fact be RNase-resistant false positives. Indeed, all these RNAs are highly structured and embedded in very proteinaceous house-keeping RNPs (87–89). Therefore, it is not possible to conclude about the localization of this group of transcripts solely based on their resistance to RNase, and orthogonal methods are required to draw the line (see below).

**Figure 4.**
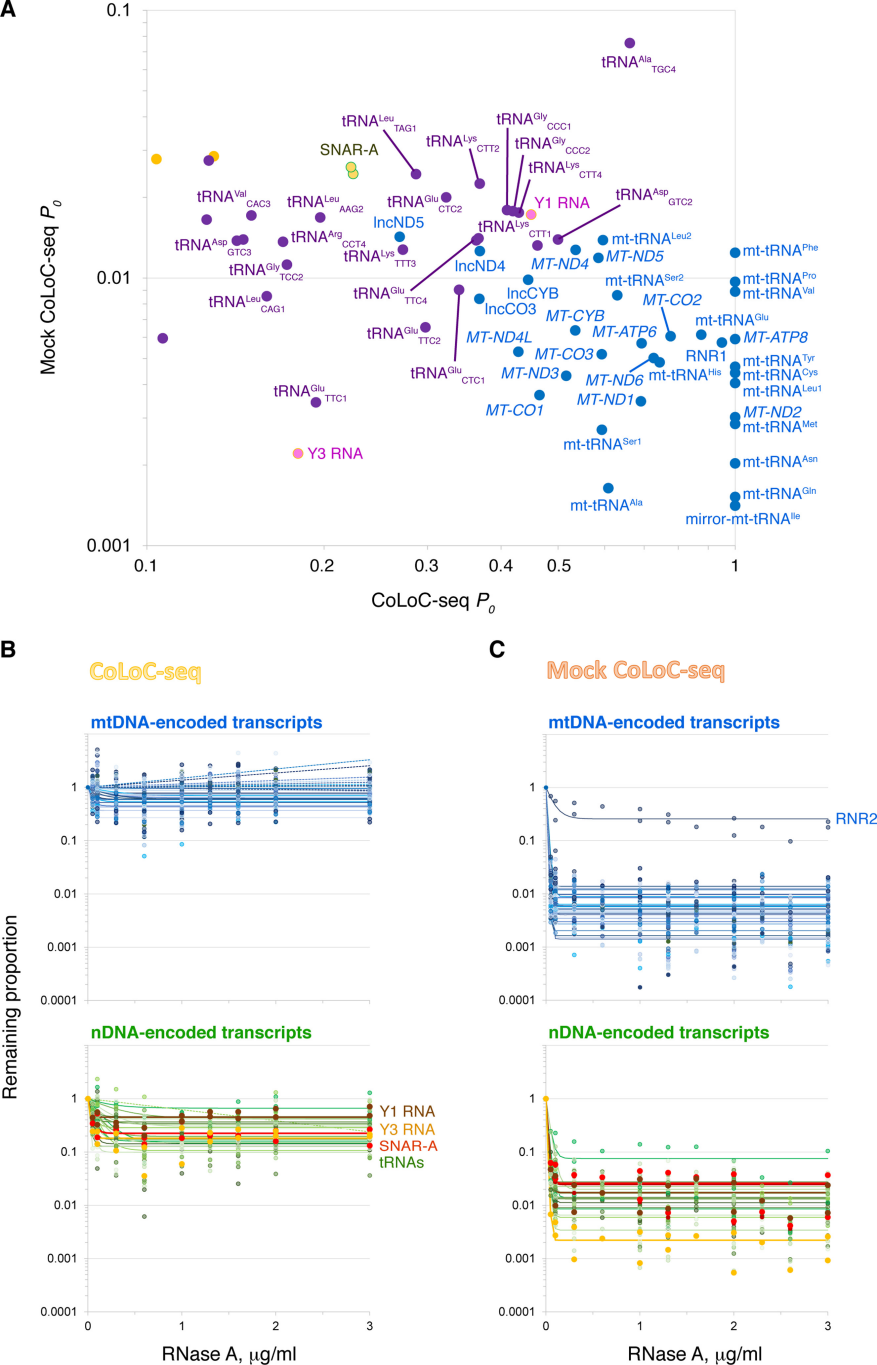
Human mitochondria-resident transcripts identified by CoLoC-seq. (**A**) Detailed view of the high CoLoC-seq *P*_0_ – low Mock CoLoC-seq *P*_0_ area from Figure 3C showing mtDNA-encoded (*blue*) and nDNA-encoded (*other colours*) transcripts specifically protected from degradation by mitochondrial membranes. (**B**, **C**) Corresponding CoLoC-seq (B) and Mock CoLoC-seq (C) profiles with fitted kinetics models indicate that these RNAs significantly resist RNase A-mediated depletion when the mitochondrial membranes are intact but get rapidly degraded once they are solubilised. Mitochondrial 16S rRNA (RNR2) is shown as an unusually RNase-resistant mtDNA-encoded transcript (see also Figure 3C and Supplementary Table S2 for the underlying data).

The most interesting part of this landscape, harbouring ∼18% of all mitochondria-associated RNAs, featured high *P*_0_ values in CoLoC-seq, where mitochondria were intact, but low *P*_0_ values in Mock CoLoC-seq, where they were lysed (Figures 3C, 4). This suggests that they are specifically protected by mitochondrial membranes and, therefore, likely represent *bona fide* resident transcripts. These RNAs include 34 mtDNA-encoded transcripts. Only one mtDNA-encoded RNA, 16S rRNA/RNR2, showed an unusually high resistance to RNase A, likely because it is embedded in the extensively proteinaceous large mitoribosomal subunit (90). Unlike proximity labelling approaches, which successfully identified long polyadenylated mtDNA-encoded RNAs but could not efficiently handle small RNA species (45–47,49), CoLoC-seq also captured 15 out of 22 mitochondrial tRNAs. This highlights its ability to interrogate this traditionally more challenging part of the transcriptome.

Strikingly, a distinct group of small PolIII-transcribed ncRNAs occupied a similar position in the plot, suggesting their mitochondrial localisation (Figure 3C and 4A). Those include the highly conserved Y1 and Y3 RNAs (91,92), the primate-specific SNAR-A RNAs (93), and a set of tRNAs. Manual inspection of their profiles confirmed that they had a significant RNase A-resistant pool in CoLoC-seq, when mitochondria were intact, but not in Mock CoLoC-seq, when they were lysed (Figure 4B, C, Supplementary Table S2). As far as the read length (75 nt) permits to see, all these RNAs appear to be full-size, untruncated transcripts (Supplementary Figure S8A–C). The extensive association of cytosolic tRNAs with human mitochondria has been previously reported (36), while the functional significance of this phenomenon remains enigmatic. The composition of the detected tRNAs is strongly biased: Glu, Asp, Lys and Gly isoacceptors overwhelmingly dominate the landscape (Supplementary Figure S8D). These tRNAs are neither the most abundant nor the least charged in HEK293 cells (94–96). However, these tRNA families are exactly those containing the smallest number of Watson-Crick face-disrupting methylations and consecutive dihydrouridines, which enables their preferential, if not exclusive, coverage in standard RNA-seq experiments (96–98). Indeed, the recent mim-tRNAseq analysis of human tRNAs (96) indicates that these tRNA families have the lowest number of modified residues causing TGIRT-mediated nucleotide misincorporation (Supplementary Figure S8E). More traditional reverse transcriptases used in RNA-seq library construction hard-stop on such positions, strongly biasing the apparent tRNA diversity. With this consideration in mind, we do not find any obvious specificity in tRNA association with mitochondria.

### Nucleus-encoded mitochondria-localised RNAs are primarily stuck in the intermembrane space

**Figure 5.**
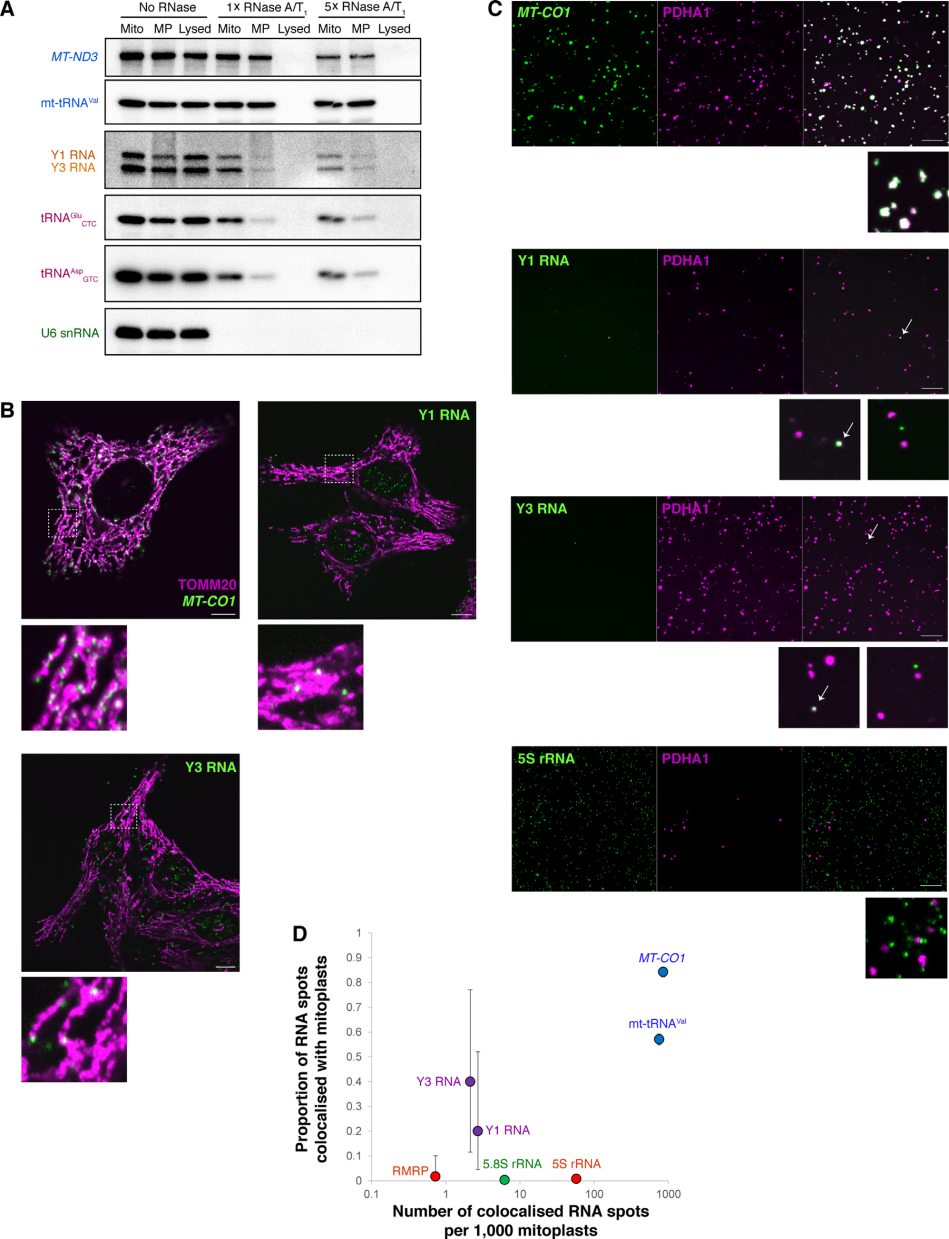
Nuclear DNA-encoded mitochondrial RNAs are scarce in the mitochondrial matrix. (**A**) Submitochondrial fractionation of mitochondria-associated transcripts. Intact mitochondria, mitoplasts obtained by osmotic disruption of the outer membrane (MP), or lysed mitochondria from HEK293 cells were treated with an RNase A/T_1_ mixture (‘1×’ corresponds to 1 μg of RNase A and 2.5 U of RNase T_1_ per mg of mitochondrial protein) or left untreated. Surviving RNAs were analysed by northern blotting with probes to select mtDNA- (*MT-ND3*, mt-tRNA^Val^) and nDNA-encoded (Y1 and Y3 RNAs, tRNA^Glu^_CTC_, tRNA^Asp^_GTC_, U6 snRNA) transcripts. (**B**) The mtDNA-encoded *MT-CO1* mRNA and two nDNA-encoded transcripts (Y1 and Y3 RNA) were detected in HepG2 cells by branched DNA smFISH and visualised under a confocal microscope (*green*). TOMM20, an outer mitochondrial membrane protein, was visualised by immunofluorescence (*magenta*). Size bar is 10 μm. Zoom-in views (10 × 10 μm) are provided for select cases of apparent colocalisation (white). See also Supplementary Figure S9A. (**C**) Mitoplasts of HEK293 cells expressing PDHA1-mCherry were prepared from crude RNase A-treated mitochondria by digitonin-mediated disruption of the outer membrane and subjected to smFISH as in panel (B). Size bar is 10 μm. Zoom-in views (10 × 10 μm) are provided for select cases of colocalisation (white, shown with arrows for Y RNAs) or of absence thereof. See also Supplementary Figure S9B, C. (**D**) Quantification of the data shown in (C). For each analysed transcript, the proportion of RNA spots colocalising with mitoplasts (±95% CI) is plotted versus the number of mitoplast-localised RNA spots per 1000 examined mitoplasts. Note that for some particularly abundant transcripts the 95% CI bars are very small.

Mitochondria have two membranes that delimit the innermost matrix subcompartment and the intermembrane space (IMS). We wondered where exactly in mitochondria the newly identified nDNA-encoded RNAs could be located. This question is important as their eventual presence in the matrix would mean that they could functionally interact with the mitochondrial genetic system. To this end, we subjected crude HEK293 mitochondria to a standard submitochondrial fractionation procedure frequently employed to interrogate protein localisation (see Materials and Methods). Intact mitochondria, mitoplasts (mitochondria with an osmotically ruptured outer membrane), or fully lysed mitochondria were treated or not with an RNase A/T_1_ mix; the remaining RNA was extracted and analysed by northern blotting. As shown in Figure 5A, mtDNA-encoded RNAs were resistant to RNase A/T_1_ digestion in both mitochondria and mitoplasts, indicating their matrix localisation. By contrast, U6 snRNA was degraded in all RNase A/T_1_-treated samples, corresponding to its extramitochondrial localisation. Interestingly, both Y RNAs and the cytosolic tRNAs we probed for remained fairly resistant to RNase A/T_1_ in intact mitochondria but were strongly depleted upon the disruption of the outer membrane. This suggests that the majority of these molecules localise to the intermembrane space, rather than to the matrix.

As an independent, *in situ* approach, we interrogated the subcellular localisation of Y RNAs using smFISH in HepG2 cells (Figure 5B). Y1 and Y3 RNAs showed both cytoplasmic and nuclear localisation in HepG2 cells, as previously reported (99,100), and accumulated to several dozen copies/cell. They were frequently found next to mitochondria and on some rare occasions (3–5 per cell) clearly colocalised with the organelles. The RNase A-resistant RMRP, which has established functions in both the nucleus and the cytoplasm (88,101–103), showed a similar localisation pattern (Supplementary Figure S9A). The mtDNA-encoded *MT-CO1* mRNA showed a nearly complete colocalisation with the mitochondrial marker (Figure 5B), whereas 5S rRNA and 5.8S rRNA were far too abundant to conclude about their degree of colocalisation (Supplementary Figure S9A), as previously reported (51).

Given the resolution limit of confocal microscopy, the observed mitochondria-associated RNAs could be either internally localised or merely attached to the surface of the organelles. To distinguish between these two possibilities, we performed smFISH on isolated mitoplasts from a HEK293 cell line expressing a matrix-localised PDHA1-mCherry marker. This cell model allows for a facile tracking of mitochondria, distinguishing them from other copurified organelles and cell debris. Additionally, the disruption of the outer membrane significantly enlarges mitoplasts, facilitating the observation of eventual colocalisation events (Supplementary Figure S9B). Importantly, the highly specific branched DNA smFISH technology, relying on joint co-binding of at least two probes and *in situ* signal amplification, permits the detection of single transcripts, independently of their length (71,72). Figure 5C, D and Supplementary Figure S9C show that the mtDNA-encoded *MT-CO1* and mt-tRNA^Val^ were predominantly colocalised with mitoplasts, as expected (1258/1493, or 84.3%, of detected *MT-CO1* molecules, 95% CI from 82.3% to 86%). By contrast, nearly all detected 5S rRNA and 5.8S rRNA molecules lay outside the mitoplasts. The same was true for the less-abundant RMRP: the few spots observed in the mitoplast preparations hardly ever colocalised with mitoplasts (1/57 occurences, 95% CI from 0% to 10.2%, Supplementary Figure S9C). Interestingly, both Y1 and Y3 RNAs could be detected either as stand-alone particles or as mitoplast-localised transcripts. However, the number of such events was extremely small, especially in comparison with mtDNA-encoded RNAs: only 10 Y1 RNA molecules were observed per 739 examined mitoplasts, 2 of which colocalised with mitoplasts (95% CI from 4.6% to 52.1%); and out of 5 detected Y3 RNA molecules (per 935 mitoplasts), 2 showed colocalisation (95% CI from 11.6% to 77.1%). Given their scarcity, assuming a Poisson distribution, these spots likely correspond to single Y RNAs. Thereby, in comparison with the mtDNA-encoded *MT-CO1* mRNA, this effectively sets the higher limit of their abundance at <1 per 300 mtDNA-encoded transcripts (taking the hyper-conservative approach assigning one mtDNA-encoded RNA to one observed mitoplast; a more realistic estimate, accounting for its higher real abundance and the fact that the *MT-CO1* mRNA is but one of many mtDNA-encoded transcripts, is likely 3–4 orders of magnitude lower). Altogether, our data suggest that, while some nDNA-encoded RNAs seem to be present in human mitochondria, their abundance in the matrix is extremely low.

### Y RNA-associated Ro60 protein is found in mitochondria but does not interact with PNPase

**Figure 6.**
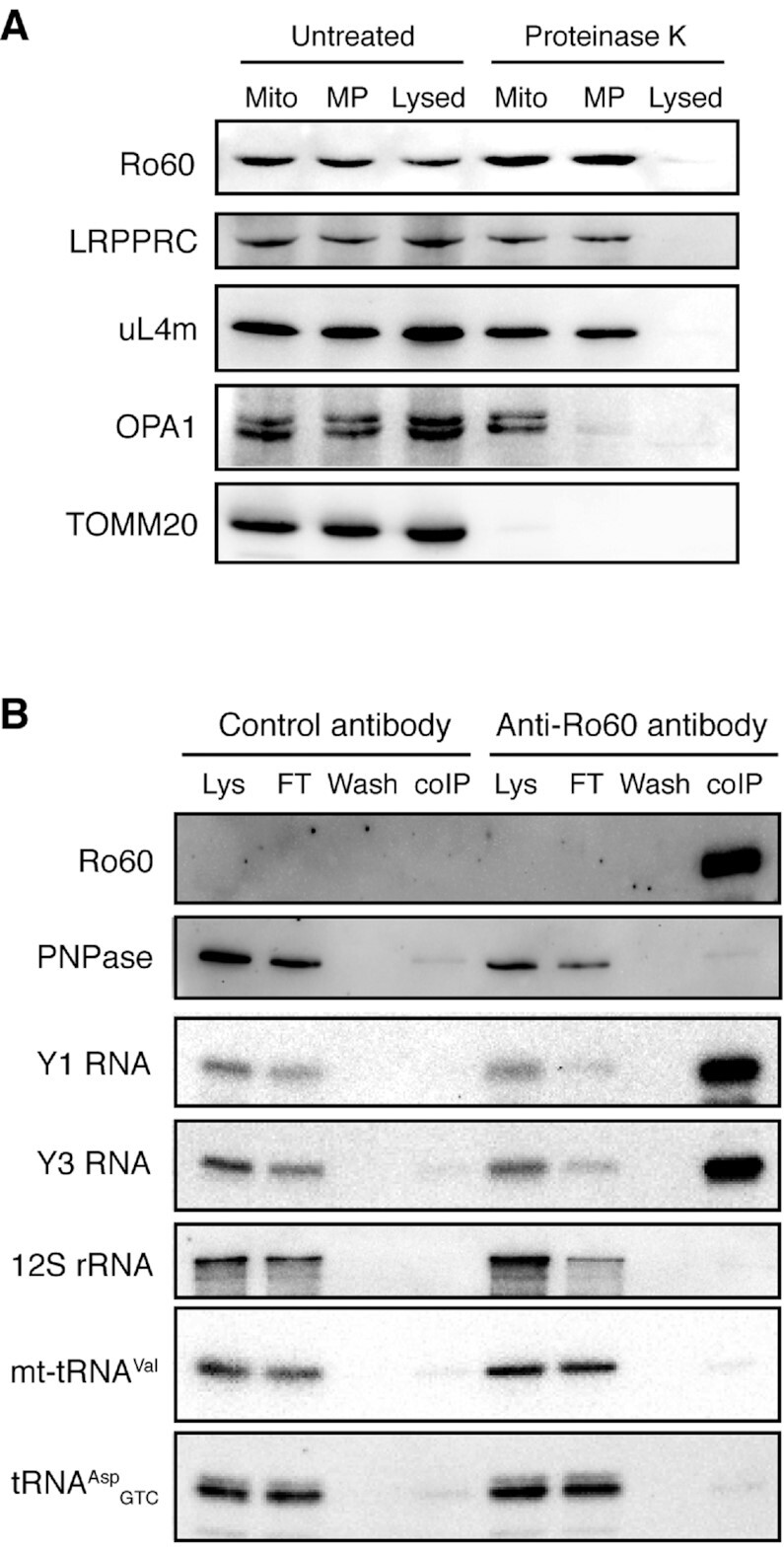
Ro60 is likely present in human mitochondria. (**A**) Submitochondrial fractionation of mitochondria-associated proteins. Intact mitochondria, mitoplasts (MP), or lysed mitochondria, obtained from HEK293 cells as in Fig. 5A, were treated with proteinase K or left untreated. Surviving proteins were analysed by western blotting. LRPPRC and uL4m are matrix proteins. OPA1 is an inner membrane protein exposed into the IMS. TOMM20 is an outer membrane protein facing the cytosol. Ro60 was detected with a mouse monoclonal antibody (Santa Cruz Biotechnology, sc-100844). See also Supplementary Figure S10B for a replicate experiment using a different anti-Ro60 antibody. (**B**) Ro60 was immunoprecipitated from crude HEK293 mitochondria, and the coimmunoprecipitates were analysed by western blotting (Ro60, PNPase) or northern blotting. ‘Lys’ – mitochondrial lysate, ‘FT’ – flow-through. Note that the Ro60 level in the lysate was below the detection limit; it could only be seen in the strongly enriched specific coIP fraction.

Finding Y RNAs in mitochondria was unexpected and made us wonder whether their key protein partner Ro60, which is required for their stability (91,104), may similarly be found in this compartment. Because Ro60 is abundant (92), it is difficult to judge about its possible association with the mitochondria by immunofluorescence (Supplementary Figure S10A). Therefore, we performed the same submitochondrial fractionation analysis, as in the case of RNAs (Figure 5A), but using resistance to proteinase K digestion as a probe for the differential submitochondrial localisation of proteins (Figure 6A and Supplementary Figure S10B). The known mitochondrial matrix-localised proteins LRPPRC and the mitoribosomal protein uL4m remained resistant to proteinase K in all samples but the lysed one. The inner membrane-embedded, IMS-exposed OPA1 protein was protected in intact mitochondria but digested upon the outer membrane rupture. The cytosol-exposed TOMM20 protein was sensitive to proteinase K in all samples (105). Strikingly, Ro60, independently identified with two different antibodies, behaved similarly to LRPPRC and uL4m (Figure 6A and Supplementary Figure S10B). Moreover, an α-Ro60 antibody coimmunoprecipitated several mitochondrial proteins from total HEK293 cell lysates (Supplementary Figure S10C). This suggests that it may indeed be present within the interior space of mitochondria along with its Y RNA ligands.

Ro60 and Y RNAs were found to form a stable RYPER complex with PNPase, which helps to degrade structured RNA molecules in *Deinococcus radiodurans* and *Salmonella enterica* (106). Since the human PNPase orthologue has been reported to localise in the mitochondrial IMS and matrix (107,108) (Supplementary Figure S10B), we wondered whether a similar assembly may exist in human cells. To this end, we immunoprecipitated Ro60 from crude HEK293 mitochondria and found that it stably interacted with both Y RNAs but not with PNPase (Figure 6B), ruling out the formation of a stable RYPER-like complex in humans.

## DISCUSSION

Fractionation-based techniques remain the most accessible and widely used subcellular transcriptomics approaches due to their simplicity, the easy handling of organelles even from genetically intractable, non-model organisms and tissues, and a high amount of material, permitting to obviate rRNA depletion and poly(A) enrichment and profile potentially all RNA classes. At the same time, their false-positive calling rate remains prohibitively high to reliably judge about the topology of RNA localisation. Here we presented a more sophisticated, topology-aware, and kinetically controlled CoLoC-seq method that corrects for false-positive identifications due to slow degradation and flags intrinsically RNase-resistant transcripts, which may be potentially false-positive. The general reaction model used by CoLoC-seq permits to robustly identify the transcripts residing inside membrane-bounded organelles, such as mitochondria. This question of localisation topology is of high importance for our understanding of how complex genetic systems operate and, in the case of the mitochondria, to what extent nDNA-encoded RNAs may intervene into post-transcriptional processes in the organelles.

By identifying nearly all mtDNA-encoded long coding and noncoding RNAs as resident, CoLoC-seq compares favourably with most advanced proximity labelling approaches, such as APEX-RIP, APEX-seq and CAP-seq, previously applied to the same model (45–47,49). Moreover, similarly to these techniques, CoLoC-seq did not find evidence for mitochondrial localisation of nDNA-encoded mRNAs or lncRNAs in HEK293 cells. By contrast, CoLoC-seq significantly outperforms the proximity labelling approaches in handling relatively short transcripts: it confidently identified 15 out of 22 mt-tRNAs (for comparison, the best performance, shown by APEX-RIP, was only 3 mt-tRNAs - (45)). Currently, only two proximity labelling methods, Halo-seq (109) and uridylation-mediated RNA recording (50), have achieved a similar degree of sensitivity for shorter RNAs (such as tRNAs). However, due to limitations imposed by its chemistry, Halo-seq cannot deal with mitochondria-localised transcripts, which are naturally oxidised due to their proximity to the electron-transport chain, while the performance of the RNA recording has yet to be tested in the mitochondrial interior.

The unique ability of CoLoC-seq to interrogate, in an unbiased way, shorter RNAs enabled us to address a long-standing and controversial question of the localisation topology of nDNA-encoded transcripts in mammalian mitochondria (51,76). For instance, although a few spliceosomal components had been detected in mitochondrial preparations (110), CoLoC-seq clearly classified them as surface-attached, even though highly abundant, contaminants (Figure 3), ruling out the existence of a splicing machinery in these organelles. A trickier and thornier case is the much-debated presence in human mitochondria of 5S rRNA, RMRP and RPPH1 RNAs (51,76). By comparing the behaviour of these transcripts in CoLoC-seq and Mock CoLoC-seq, we found that they were remarkably recalcitrant to RNase A degradation even when there were no membranes to protect them (Figures 2, 3 and Supplementary Figure S1). Direct microscopy observation of 5S rRNA and RMRP in preparations of RNase-treated mitoplasts confirmed that these RNAs were present nearly exclusively in extra-mitochondrial material (Figure 5C), suggesting that they were protected from degradation by other means, e.g. associated RNA-binding proteins. This finding cautions against such false-positive cases, which, however rare, cannot be easily spotted in simpler fractionation-based experimental setups and require cross-validation by orthogonal approaches. Of note, the extensive protection of RNAs by proteins (111–113) or by intricate structure (114) has been recognised as a major challenge in the extracellular RNA field, which more recently urged the community to reconsider the localisation of exported RNAs within free ribonucleoproteins (RNPs) versus extracellular vesicles (16,17,38,40–42,115–119).

We detected cytosolic tRNAs, SNAR-A and Y RNAs as significantly mitochondria-associated. With their primary localisation to the IMS, the functional significance of their mitochondrial destination is unclear. SNAR-A are poorly characterised primate-specific PolIII transcripts, normally found in the cytoplasm in association with cytosolic ribosomes (93). Y RNAs, stably associated with the deeply conserved Ro60 protein, are much better studied and form RNPs involved in RNA quality control and turnover (91). Given that they are present in 5% bacteria, where they team up with proteins like PNPase to better degrade structured RNAs (106,120), their finding in the mitochondria probably should not appear so surprising. However, despite their apparent colocation (in the IMS and/or the matrix), we do not find evidence for a stable Y RNA-Ro60-PNPase complex in human cells. More importantly, although the detection of Y RNAs inside isolated RNase-treated mitoplasts supports their low-level presence in the matrix, their estimated copy number in this compartment is extremely low. The mitochondrial and nuclear RNA worlds thus appear largely separated in human cells.

CoLoC-seq has been designed based on basic biochemical principles and must in principle be applicable to any membrane-bounded or, more specifically, RNase-impermeable entities. Among them, genome-containing organelles, such as chloroplasts and apicoplasts, but also organelle-like endosymbionts and essentially all enveloped and even some non-enveloped viruses, are all objects of highest interest, as their ability to import extraneous RNA, which may have important functional and evolutionary implications, has not been systematically evaluated. Similarly, robust profiling of the RNA content of extracellular vesicles from both eukaryotic and bacterial sources remains problematic in terms of localisation topology and may require an adapted CoLoC-seq pipeline, e.g. incorporating a proteinase K pre-treatment step to expose RNP-embedded contaminants (112,115–117).

### Limitations of CoLoC-seq

As for now, CoLoC-seq did not explicitly profile very short transcripts, such as miRNAs. Therefore, their possible localisation inside the human mitochondria remains unanswered. It will likely require a number of significant technical tweaks which are beyond the scope of this paper. A major hurdle in this direction is the nearly complete shielding of miRNAs by the associated Ago proteins (112) which, compounded by a generally lower susceptibility of miRNAs to degradation (62), will yet have to be dealt with by essentially all subcellular transcriptomics approaches. In a similar vein, the observed tRNA diversity was likely affected by the extensive modification of many cytosolic and some mtDNA-encoded tRNAs, which can be improved with existing demethylating and TGIRT-dependent tRNA-seq strategies (96,97,121,122). Due to the specific sequencing protocol, CoLoC-seq is naturally blind to regular 5’-OH and 2’/3’-phosphorylated RNA species (e.g. tRNA fragments) and to circRNAs. However, those can still be visualised and quantified by northern blotting. Finally, one may expect that CoLoC-seq might have missed some low-abundance RNAs that could still be localised in the mitochondria. However, the copy number estimates for mitochondria-localised Y RNAs enabled by smFISH in isolated mitoplasts (Figure 5C) suggest that we have reached the limit of biologically relevant detection, and the existence of further such transcripts is unlikely.

## DATA AVAILABILITY

Scripts used for alignment, coverage calculation, and feature quantification of CoLoC-seq data can be found at Zenodo (https://doi.org/10.5281/zenodo.6389451). The CoLoC-seq and Mock-CoLoC-seq data presented in this article have been deposited in NCBI Gene Expression Omnibus (GEO) under accession number GSE183167 [link to access the dataset https://www.ncbi.nlm.nih.gov/geo/query/acc.cgi?acc=GSE183167]. The mass spectrometry proteomics data have been deposited in open access to the ProteomeXchange Consortium via the PRIDE partner repository with the dataset identifier PXD037383.

## Supplementary Material

gkac1183_Supplemental_Files

## References

[1] Quirós P.M. , Mottis A., Auwerx J. Mitonuclear communication in homeostasis and stress. Nat. Rev. Mol. Cell Biol. 2016; 17:213–226. 26956194 10.1038/nrm.2016.23

[2] Richter-Dennerlein R. , Oeljeklaus S., Lorenzi I., Ronsör C., Bareth B., Schendzielorz A.B., Wang C., Warscheid B., Rehling P., Dennerlein S. Mitochondrial protein synthesis adapts to influx of nuclear-encoded protein. Cell. 2016; 167:471–483. 27693358 10.1016/j.cell.2016.09.003 PMC5055049

[3] Couvillion M.T. , Soto I.C., Shipkovenska G., Churchman L.S. Synchronized mitochondrial and cytosolic translation programs. Nature. 2016; 533:499–503. 27225121 10.1038/nature18015 PMC4964289

[4] Sloan K.E. , Gleizes P.-E., Bohnsack M.T. Nucleocytoplasmic transport of RNAs and RNA-protein complexes. J. Mol. Biol. 2016; 428:2040–2059. 26434509 10.1016/j.jmb.2015.09.023

[5] Massenet S. , Bertrand E., Verheggen C. Assembly and trafficking of box C/D and H/ACA snoRNPs. RNA Biol. 2017; 14:680–692. 27715451 10.1080/15476286.2016.1243646 PMC5519232

[6] Chatterjee K. , Nostramo R.T., Wan Y., Hopper A.K. tRNA dynamics between the nucleus, cytoplasm and mitochondrial surface: location, location, location. Biochim. Biophys. Acta Gene Regul. Mech. 2018; 1861:373–386. 29191733 10.1016/j.bbagrm.2017.11.007 PMC5882565

[7] Ozata D.M. , Gainetdinov I., Zoch A., O’Carroll D., Zamore P.D. PIWI-interacting RNAs: small RNAs with big functions. Nat. Rev. Genet. 2019; 20:89–108. 30446728 10.1038/s41576-018-0073-3

[8] Williams T. , Ngo L.H., Wickramasinghe V.O. Nuclear export of RNA: different sizes, shapes and functions. Semin. Cell Dev. Biol. 2018; 75:70–77. 28866329 10.1016/j.semcdb.2017.08.054

[9] Lashkevich K.A. , Dmitriev S.E. mRNA targeting, transport and local translation in eukaryotic cells: from the classical view to a diversity of new concepts. Mol. Biol. 2021; 55:507–537. 34092811 10.1134/S0026893321030080 PMC8164833

[10] Gasparski A.N. , Mason D.E., Moissoglu K., Mili S. Regulation and outcomes of localized RNA translation. Wiley Interdiscip. Rev. RNA. 2022; 13:e1721. 35166036 10.1002/wrna.1721 PMC9787767

[11] Béthune J. , Jansen R.-P., Feldbrügge M., Zarnack K. Membrane-associated RNA-binding proteins orchestrate organelle-coupled translation. Trends Cell Biol. 2019; 29:178–188. 30455121 10.1016/j.tcb.2018.10.005

[12] Engel K.L. , Arora A., Goering R., Lo H.-Y.G., Taliaferro J.M. Mechanisms and consequences of subcellular RNA localization across diverse cell types. Traffic. 2020; 21:404–418. 32291836 10.1111/tra.12730 PMC7304542

[13] Gehrke S. , Wu Z., Klinkenberg M., Sun Y., Auburger G., Guo S., Lu B. PINK1 and Parkin control localized translation of respiratory chain component mRNAs on mitochondria outer membrane. Cell Metab. 2015; 21:95–108. 25565208 10.1016/j.cmet.2014.12.007 PMC4455944

[14] Wang E.T. , Taliaferro J.M., Lee J.-A., Sudhakaran I.P., Rossoll W., Gross C., Moss K.R., Bassell G.J. Dysregulation of mRNA localization and translation in genetic disease. J. Neurosci. 2016; 36:11418–11426. 27911744 10.1523/JNEUROSCI.2352-16.2016 PMC5125209

[15] Bovaird S. , Patel D., Padilla J.-C.A., Lécuyer E. Biological functions, regulatory mechanisms, and disease relevance of RNA localization pathways. FEBS Lett. 2018; 592:2948–2972. 30132838 10.1002/1873-3468.13228

[16] Gruner H.N. , McManus M.T. Examining the evidence for extracellular RNA function in mammals. Nat. Rev. Genet. 2021; 22:448–458. 33824487 10.1038/s41576-021-00346-8

[17] Tosar J.P. , Witwer K., Cayota A. Revisiting extracellular RNA release, processing, and function. Trends Biochem. Sci. 2021; 46:438–445. 33413996 10.1016/j.tibs.2020.12.008 PMC8122015

[18] Dauros-Singorenko P. , Blenkiron C., Phillips A., Swift S. The functional RNA cargo of bacterial membrane vesicles. FEMS Microbiol. Lett. 2018; 365:10.1093/femsle/fny023. 29390056

[19] Lécrivain A.-L. , Beckmann B.M. Bacterial RNA in extracellular vesicles: a new regulator of host-pathogen interactions?. Biochim. Biophys. Acta Gene Regul. Mech. 2020; 1863:194519. 32142907 10.1016/j.bbagrm.2020.194519

[20] Lefebvre F.A. , Lécuyer E. Small luggage for a long journey: transfer of vesicle-enclosed small RNA in interspecies communication. Front. Microbiol. 2017; 8:377. 28360889 10.3389/fmicb.2017.00377 PMC5352665

[21] Bresnahan W.A. , Shenk T. A subset of viral transcripts packaged within human cytomegalovirus particles. Science. 2000; 288:2373–2376. 10875924 10.1126/science.288.5475.2373

[22] Sciortino M.T. , Suzuki M., Taddeo B., Roizman B. RNAs extracted from herpes simplex virus 1 virions: apparent selectivity of viral but not cellular RNAs packaged in virions. J. Virol. 2001; 75:8105–8116. 11483756 10.1128/JVI.75.17.8105-8116.2001 PMC115055

[23] Onafuwa-Nuga A.A. , Telesnitsky A., King S.R. 7SL RNA, but not the 54-kd signal recognition particle protein, is an abundant component of both infectious HIV-1 and minimal virus-like particles. RNA. 2006; 12:542–546. 16489186 10.1261/rna.2306306 PMC1421090

[24] Lin X. , Li X., Liang D., Lan K. MicroRNAs and unusual small RNAs discovered in Kaposi's sarcoma-associated herpesvirus virions. J. Virol. 2012; 86:12717–12730. 22973026 10.1128/JVI.01473-12 PMC3497663

[25] Brameier M. , Ibing W., Höfer K., Montag J., Stahl-Hennig C., Motzkus D. Mapping the small RNA content of simian immunodeficiency virions (SIV). PLoS One. 2013; 8:e75063. 24086438 10.1371/journal.pone.0075063 PMC3781035

[26] Liu Y.-T. , Strugatsky D., Liu W., Zhou Z.H. Structure of human cytomegalovirus virion reveals host tRNA binding to capsid-associated tegument protein pp150. Nat. Commun. 2021; 12:5513. 34535641 10.1038/s41467-021-25791-1 PMC8448752

[27] Taliaferro J.M. , Wang E.T., Burge C.B. Genomic analysis of RNA localization. RNA Biol. 2014; 11:1040–1050. 25483039 10.4161/rna.32146 PMC4615561

[28] Christopher J.A. , Geladaki A., Dawson C.S., Vennard O.L., Lilley K.S. Subcellular transcriptomics and proteomics: a comparative methods review. Mol. Cell Proteomics. 2022; 21:100186. 34922010 10.1016/j.mcpro.2021.100186 PMC8864473

[29] Chen K.H. , Boettiger A.N., Moffitt J.R., Wang S., Zhuang X. RNA imaging. Spatially resolved, highly multiplexed RNA profiling in single cells. Science. 2015; 348:aaa6090. 25858977 10.1126/science.aaa6090 PMC4662681

[30] Xia C. , Fan J., Emanuel G., Hao J., Zhuang X. Spatial transcriptome profiling by MERFISH reveals subcellular RNA compartmentalization and cell cycle-dependent gene expression. Proc. Natl. Acad. Sci. U.S.A. 2019; 116:19490–19499. 31501331 10.1073/pnas.1912459116 PMC6765259

[31] Shah S. , Takei Y., Zhou W., Lubeck E., Yun J., Eng C.-H.L., Koulena N., Cronin C., Karp C., Liaw E.J. et al. Dynamics and spatial genomics of the nascent transcriptome by intron seqFISH. Cell. 2018; 174:363–376. 29887381 10.1016/j.cell.2018.05.035 PMC6046268

[32] Li Y. , Ke K., Spitale R.C. Biochemical methods to image and analyze RNA localization: from one to many. Biochemistry. 2019; 58:379–386. 30444114 10.1021/acs.biochem.8b01087

[33] Fazal F.M. , Chang H.Y. Subcellular spatial transcriptomes: emerging frontier for understanding gene regulation. Cold Spring Harb. Symp. Quant. Biol. 2019; 84:31–45. 32482897 10.1101/sqb.2019.84.040352 PMC7426137

[34] Greijer A.E. , Dekkers C.A., Middeldorp J.M. Human cytomegalovirus virions differentially incorporate viral and host cell RNA during the assembly process. J. Virol. 2000; 74:9078–9082. 10982353 10.1128/jvi.74.19.9078-9082.2000 PMC102105

[35] Cliffe A.R. , Nash A.A., Dutia B.M. Selective uptake of small RNA molecules in the virion of murine gammaherpesvirus 68. J. Virol. 2009; 83:2321–2326. 19109392 10.1128/JVI.02303-08 PMC2643712

[36] Mercer T.R. , Neph S., Dinger M.E., Crawford J., Smith M.A., Shearwood A.-M.J., Haugen E., Bracken C.P., Rackham O., Stamatoyannopoulos J.A. et al. The human mitochondrial transcriptome. Cell. 2011; 146:645–658. 21854988 10.1016/j.cell.2011.06.051 PMC3160626

[37] Valadi H. , Ekström K., Bossios A., Sjöstrand M., Lee J.J., Lötvall J.O. Exosome-mediated transfer of mRNAs and microRNAs is a novel mechanism of genetic exchange between cells. Nat. Cell Biol. 2007; 9:654–659. 17486113 10.1038/ncb1596

[38] Tosar J.P. , Gámbaro F., Sanguinetti J., Bonilla B., Witwer K.W., Cayota A. Assessment of small RNA sorting into different extracellular fractions revealed by high-throughput sequencing of breast cell lines. Nucleic Acids Res. 2015; 43:5601–5616. 25940616 10.1093/nar/gkv432 PMC4477662

[39] Shurtleff M.J. , Yao J., Qin Y., Nottingham R.M., Temoche-Diaz M.M., Schekman R., Lambowitz A.M. Broad role for YBX1 in defining the small noncoding RNA composition of exosomes. Proc. Natl. Acad. Sci. U.S.A. 2017; 114:E8987–E8995. 29073095 10.1073/pnas.1712108114 PMC5663387

[40] Murillo O.D. , Thistlethwaite W., Rozowsky J., Subramanian S.L., Lucero R., Shah N., Jackson A.R., Srinivasan S., Chung A., Laurent C.D. et al. exRNA atlas analysis reveals distinct extracellular RNA cargo types and their carriers present across human biofluids. Cell. 2019; 177:463–477. 30951672 10.1016/j.cell.2019.02.018 PMC6616370

[41] Jeppesen D.K. , Fenix A.M., Franklin J.L., Higginbotham J.N., Zhang Q., Zimmerman L.J., Liebler D.C., Ping J., Liu Q., Evans R. et al. Reassessment of exosome composition. Cell. 2019; 177:428–445. 30951670 10.1016/j.cell.2019.02.029 PMC6664447

[42] Srinivasan S. , Yeri A., Cheah P.S., Chung A., Danielson K., De Hoff P., Filant J., Laurent C.D., Laurent L.D., Magee R. et al. Small RNA sequencing across diverse biofluids identifies optimal methods for exRNA isolation. Cell. 2019; 177:446–462. 30951671 10.1016/j.cell.2019.03.024 PMC6557167

[43] Jan C.H. , Williams C.C., Weissman J.S. Principles of ER cotranslational translocation revealed by proximity-specific ribosome profiling. Science. 2014; 346:1257521. 25378630 10.1126/science.1257521 PMC4285348

[44] Williams C.C. , Jan C.H., Weissman J.S. Targeting and plasticity of mitochondrial proteins revealed by proximity-specific ribosome profiling. Science. 2014; 346:748–751. 25378625 10.1126/science.1257522 PMC4263316

[45] Kaewsapsak P. , Shechner D.M., Mallard W., Rinn J.L., Ting A.Y. Live-cell mapping of organelle-associated RNAs via proximity biotinylation combined with protein-RNA crosslinking. Elife. 2017; 6:e29224. 29239719 10.7554/eLife.29224 PMC5730372

[46] Fazal F.M. , Han S., Parker K.R., Kaewsapsak P., Xu J., Boettiger A.N., Chang H.Y., Ting A.Y. Atlas of subcellular RNA localization revealed by APEX-seq. Cell. 2019; 178:473–490. 31230715 10.1016/j.cell.2019.05.027 PMC6786773

[47] Zhou Y. , Wang G., Wang P., Li Z., Yue T., Wang J., Zou P. Expanding APEX2 substrates for proximity-dependent labeling of nucleic acids and proteins in living cells. Angew. Chem. Int. Ed. Engl. 2019; 58:11763–11767. 31240809 10.1002/anie.201905949

[48] Benhalevy D. , Anastasakis D.G., Hafner M. Proximity-CLIP provides a snapshot of protein-occupied RNA elements in subcellular compartments. Nat. Methods. 2018; 15:1074–1082. 30478324 10.1038/s41592-018-0220-y PMC6289640

[49] Wang P. , Tang W., Li Z., Zou Z., Zhou Y., Li R., Xiong T., Wang J., Zou P. Mapping spatial transcriptome with light-activated proximity-dependent RNA labeling. Nat. Chem. Biol. 2019; 15:1110–1119. 31591565 10.1038/s41589-019-0368-5

[50] Medina-Munoz H.C. , Lapointe C.P., Porter D.F., Wickens M. Records of RNA locations in living yeast revealed through covalent marks. Proc. Natl. Acad. Sci. U.S.A. 2020; 117:23539–23547. 32907940 10.1073/pnas.1921408117 PMC7519331

[51] Jeandard D. , Smirnova A., Tarassov I., Barrey E., Smirnov A., Entelis N. Import of non-coding RNAs into human mitochondria: a critical review and emerging approaches. Cells. 2019; 8:286. 30917553 10.3390/cells8030286 PMC6468882

[52] Xing L. , Tikoo S.K. Viral RNAs detected in virions of porcine adenovirus type 3. Virology. 2004; 321:372–382. 15051396 10.1016/j.virol.2003.12.025

[53] Cho T.-J. , Dreher T.W. Encapsidation of genomic but not subgenomic Turnip yellow mosaic virus RNA by coat protein provided in trans. Virology. 2006; 356:126–135. 16942786 10.1016/j.virol.2006.06.038

[54] Routh A. , Domitrovic T., Johnson J.E. Host RNAs, including transposons, are encapsidated by a eukaryotic single-stranded RNA virus. Proc. Natl. Acad. Sci. U.S.A. 2012; 109:1907–1912. 22308402 10.1073/pnas.1116168109 PMC3277583

[55] Padrón A. , Iwasaki S., Ingolia N.T. Proximity RNA labeling by APEX-seq reveals the organization of translation initiation complexes and repressive RNA granules. Mol. Cell. 2019; 75:875–887. 31442426 10.1016/j.molcel.2019.07.030 PMC6834362

[56] Dussurget O. , Roulland-Dussoix D. Rapid, sensitive PCR-based detection of mycoplasmas in simulated samples of animal sera. Appl. Environ. Microbiol. 1994; 60:953–959. 8161186 10.1128/aem.60.3.953-959.1994 PMC201416

[57] Entelis N.S. , Kolesnikova O.A., Dogan S., Martin R.P., Tarassov I.A. 5 S rRNA and tRNA import into human mitochondria. Comparison of in vitro requirements. J. Biol. Chem. 2001; 276:45642–45653. 11551911 10.1074/jbc.M103906200

[58] Ekstrand M.I. Mitochondrial transcription factor A regulates mtDNA copy number in mammals. Hum. Mol. Genet. 2004; 13:935–944. 15016765 10.1093/hmg/ddh109

[59] Comte C. , Tonin Y., Heckel-Mager A.-M., Boucheham A., Smirnov A., Auré K., Lombès A., Martin R.P., Entelis N., Tarassov I. Mitochondrial targeting of recombinant RNAs modulates the level of a heteroplasmic mutation in human mitochondrial DNA associated with Kearns Sayre Syndrome. Nucleic Acids Res. 2013; 41:418–433. 23087375 10.1093/nar/gks965 PMC3592399

[60] Dovydenko I. , Heckel A.-M., Tonin Y., Gowher A., Venyaminova A., Tarassov I., Entelis N. Mitochondrial targeting of recombinant RNA. Methods Mol. Biol. 2015; 1265:209–225. 25634278 10.1007/978-1-4939-2288-8_16

[61] Loutre R. , Heckel A.-M., Smirnova A., Entelis N., Tarassov I. Can mitochondrial DNA be CRISPRized: pro and contra. IUBMB Life. 2018; 70:1233–1239. 30184317 10.1002/iub.1919

[62] Aryani A. , Denecke B. In vitro application of ribonucleases: comparison of the effects on mRNA and miRNA stability. BMC Res. Notes. 2015; 8:164. 25899823 10.1186/s13104-015-1114-z PMC4411928

[63] Yang W. Nucleases: diversity of structure, function and mechanism. Q Rev. Biophys. 2011; 44:1–93. 20854710 10.1017/S0033583510000181 PMC6320257

[64] Summer S. , Smirnova A., Gabriele A., Toth U., Fasemore A.M., Förstner K.U., Kuhn L., Chicher J., Hammann P., Mitulović G. et al. YBEY is an essential biogenesis factor for mitochondrial ribosomes. Nucleic Acids Res. 2020; 48:9762–9786. 32182356 10.1093/nar/gkaa148 PMC7515705

[65] Martin M. Cutadapt removes adapter sequences from high-throughput sequencing reads. EMBnet j. 2011; 17:10.

[66] Förstner K.U. , Vogel J., Sharma C.M. READemption–a tool for the computational analysis of deep-sequencing-based transcriptome data. Bioinformatics. 2014; 30:3421–3423. 25123900 10.1093/bioinformatics/btu533

[67] O’Leary N.A. , Wright M.W., Brister J.R., Ciufo S., Haddad D., McVeigh R., Rajput B., Robbertse B., Smith-White B., Ako-Adjei D. et al. Reference sequence (RefSeq) database at NCBI: current status, taxonomic expansion, and functional annotation. Nucleic Acids Res. 2016; 44:D733–D745. 26553804 10.1093/nar/gkv1189 PMC4702849

[68] Otto C. , Stadler P.F., Hoffmann S. Lacking alignments? The next-generation sequencing mapper segemehl revisited. Bioinformatics. 2014; 30:1837–1843. 24626854 10.1093/bioinformatics/btu146

[69] Park C. , Raines R.T. Catalysis by ribonuclease A is limited by the rate of substrate association. Biochemistry. 2003; 42:3509–3518. 12653555 10.1021/bi026076k

[70] Jourdain A.A. , Koppen M., Wydro M., Rodley C.D., Lightowlers R.N., Chrzanowska-Lightowlers Z.M., Martinou J.-C. GRSF1 regulates RNA processing in mitochondrial RNA granules. Cell Metab. 2013; 17:399–410. 23473034 10.1016/j.cmet.2013.02.005 PMC3593211

[71] Smirnova A. , Richert L., Smirnov A., Mély Y., Tarassov I. Suborganellar localization of mitochondrial proteins and transcripts in human cells. Methods Mol. Biol. 2021; 2277:157–173. 34080151 10.1007/978-1-0716-1270-5_11

[72] Battich N. , Stoeger T., Pelkmans L. Image-based transcriptomics in thousands of single human cells at single-molecule resolution. Nat. Methods. 2013; 10:1127–1133. 24097269 10.1038/nmeth.2657

[73] Rizk A. , Paul G., Incardona P., Bugarski M., Mansouri M., Niemann A., Ziegler U., Berger P., Sbalzarini I.F. Segmentation and quantification of subcellular structures in fluorescence microscopy images using Squassh. Nat. Protoc. 2014; 9:586–596. 24525752 10.1038/nprot.2014.037

[74] Schindelin J. , Arganda-Carreras I., Frise E., Kaynig V., Longair M., Pietzsch T., Preibisch S., Rueden C., Saalfeld S., Schmid B. et al. Fiji: an open-source platform for biological-image analysis. Nat. Methods. 2012; 9:676–682. 22743772 10.1038/nmeth.2019 PMC3855844

[75] Gruber A.R. , Lorenz R., Bernhart S.H., Neubock R., Hofacker I.L. The Vienna RNA Websuite. Nucleic Acids Res. 2008; 36:W70–W74. 18424795 10.1093/nar/gkn188 PMC2447809

[76] Gammage P.A. , Moraes C.T., Minczuk M. Mitochondrial genome engineering: the revolution may not be CRISPR-Ized. Trends Genet. 2018; 34:101–110. 29179920 10.1016/j.tig.2017.11.001 PMC5783712

[77] Yoshionari S. , Koike T., Yokogawa T., Nishikawa K., Ueda T., Miura K., Watanabe K. Existence of nuclear-encoded 5S-rRNA in bovine mitochondria. FEBS Lett. 1994; 338:137–142. 7508404 10.1016/0014-5793(94)80351-x

[78] Magalhães P.J. , Andreu A.L., Schon E.A. Evidence for the presence of 5S rRNA in mammalian mitochondria. Mol. Biol. Cell. 1998; 9:2375–2382. 9725900 10.1091/mbc.9.9.2375 PMC25503

[79] Wang G. , Chen H.-W., Oktay Y., Zhang J., Allen E.L., Smith G.M., Fan K.C., Hong J.S., French S.W., McCaffery J.M. et al. PNPASE regulates RNA import into mitochondria. Cell. 2010; 142:456–467. 20691904 10.1016/j.cell.2010.06.035 PMC2921675

[80] Smirnov A. , Entelis N., Martin R.P., Tarassov I. Biological significance of 5S rRNA import into human mitochondria: role of ribosomal protein MRP-L18. Genes Dev. 2011; 25:1289–1305. 21685364 10.1101/gad.624711 PMC3127430

[81] Zelenka J. , Alán L., Jabůrek M., Ježek P. Import of desired nucleic acid sequences using addressing motif of mitochondrial ribosomal 5S-rRNA for fluorescent in vivo hybridization of mitochondrial DNA and RNA. J. Bioenerg. Biomembr. 2014; 46:147–156. 24562889 10.1007/s10863-014-9543-2

[82] Autour A. , Jeng S.C.Y., Cawte A.D., Abdolahzadeh A., Galli A., Panchapakesan S.S.S., Rueda D., Ryckelynck M., Unrau P.J. Fluorogenic RNA Mango aptamers for imaging small non-coding RNAs in mammalian cells. Nat. Commun. 2018; 9:656. 29440634 10.1038/s41467-018-02993-8 PMC5811451

[83] Thompson J.E. , Kutateladze T.G., Schuster M.C., Venegas F.D., Messmore J.M., Raines R.T. Limits to catalysis by ribonuclease A. Bioorg. Chem. 1995; 23:471–481. 21799547 10.1006/bioo.1995.1033 PMC3144031

[84] Chang D.D. , Clayton D.A. A mammalian mitochondrial RNA processing activity contains nucleus-encoded RNA. Science. 1987; 235:1178–1184. 2434997 10.1126/science.2434997

[85] Puranam R.S. , Attardi G. The RNase P associated with HeLa cell mitochondria contains an essential RNA component identical in sequence to that of the nuclear RNase P. Mol. Cell Biol. 2001; 21:548–561. 11134342 10.1128/MCB.21.2.548-561.2001 PMC86618

[86] Noh J.H. , Kim K.M., Abdelmohsen K., Yoon J.-H., Panda A.C., Munk R., Kim J., Curtis J., Moad C.A., Wohler C.M. et al. HuR and GRSF1 modulate the nuclear export and mitochondrial localization of the lncRNA RMRP. Genes Dev. 2016; 30:1224–1239. 27198227 10.1101/gad.276022.115 PMC4888842

[87] Khatter H. , Myasnikov A.G., Natchiar S.K., Klaholz B.P. Structure of the human 80S ribosome. Nature. 2015; 520:640–645. 25901680 10.1038/nature14427

[88] Lan P. , Zhou B., Tan M., Li S., Cao M., Wu J., Lei M. Structural insight into precursor ribosomal RNA processing by ribonuclease MRP. Science. 2020; 369:656–663. 32586950 10.1126/science.abc0149

[89] Wu J. , Niu S., Tan M., Huang C., Li M., Song Y., Wang Q., Chen J., Shi S., Lan P. et al. Cryo-EM structure of the human ribonuclease P holoenzyme. Cell. 2018; 175:1393–1404. 30454648 10.1016/j.cell.2018.10.003

[90] Brown A. , Amunts A., Bai X.-C., Sugimoto Y., Edwards P.C., Murshudov G., Scheres S.H.W., Ramakrishnan V. Structure of the large ribosomal subunit from human mitochondria. Science. 2014; 346:718–722. 25278503 10.1126/science.1258026 PMC4246062

[91] Boccitto M. , Wolin S.L. Ro60 and Y RNAs: structure, functions, and roles in autoimmunity. Crit. Rev. Biochem. Mol. Biol. 2019; 54:133–152. 31084369 10.1080/10409238.2019.1608902 PMC6542706

[92] Leng Y. , Sim S., Magidson V., Wolin S.L. Noncoding Y RNAs regulate the levels, subcellular distribution and protein interactions of their Ro60 autoantigen partner. Nucleic Acids Res. 2020; 48:6919–6930. 32469055 10.1093/nar/gkaa414 PMC7337961

[93] Parrott A.M. , Tsai M., Batchu P., Ryan K., Ozer H.L., Tian B., Mathews M.B. The evolution and expression of the snaR family of small non-coding RNAs. Nucleic Acids Res. 2011; 39:1485–1500. 20935053 10.1093/nar/gkq856 PMC3045588

[94] Shigematsu M. , Honda S., Loher P., Telonis A.G., Rigoutsos I., Kirino Y. YAMAT-seq: an efficient method for high-throughput sequencing of mature transfer RNAs. Nucleic Acids Res. 2017; 45:e70. 28108659 10.1093/nar/gkx005 PMC5605243

[95] Evans M.E. , Clark W.C., Zheng G., Pan T. Determination of tRNA aminoacylation levels by high-throughput sequencing. Nucleic Acids Res. 2017; 45:e133. 28586482 10.1093/nar/gkx514 PMC5737633

[96] Behrens A. , Rodschinka G., Nedialkova D.D. High-resolution quantitative profiling of tRNA abundance and modification status in eukaryotes by mim-tRNAseq. Mol. Cell. 2021; 81:1802–1815. 33581077 10.1016/j.molcel.2021.01.028 PMC8062790

[97] Clark W.C. , Evans M.E., Dominissini D., Zheng G., Pan T. tRNA base methylation identification and quantification via high-throughput sequencing. RNA. 2016; 22:1771–1784. 27613580 10.1261/rna.056531.116 PMC5066629

[98] de Crécy-Lagard V. , Boccaletto P., Mangleburg C.G., Sharma P., Lowe T.M., Leidel S.A., Bujnicki J.M. Matching tRNA modifications in humans to their known and predicted enzymes. Nucleic Acids Res. 2019; 47:2143–2159. 30698754 10.1093/nar/gkz011 PMC6412123

[99] Matera A.G. , Frey M.R., Margelot K., Wolin S.L. A perinucleolar compartment contains several RNA polymerase III transcripts as well as the polypyrimidine tract-binding protein, hnRNP I. J. Cell Biol. 1995; 129:1181–1193. 7539809 10.1083/jcb.129.5.1181 PMC2120477

[100] Farris A.D. , Puvion-Dutilleul F., Puvion E., Harley J.B., Lee L.A. The ultrastructural localization of 60-kDa Ro protein and human cytoplasmic RNAs: association with novel electron-dense bodies. Proc. Natl. Acad. Sci. U.S.A. 1997; 94:3040–3045. 9096342 10.1073/pnas.94.7.3040 PMC20318

[101] Goldfarb K.C. , Cech T.R. Targeted CRISPR disruption reveals a role for RNase MRP RNA in human preribosomal RNA processing. Genes Dev. 2017; 31:59–71. 28115465 10.1101/gad.286963.116 PMC5287113

[102] Park O.H. , Ha H., Lee Y., Boo S.H., Kwon D.H., Song H.K., Kim Y.K. Endoribonucleolytic cleavage of m^6^A-containing RNAs by RNase P/MRP complex. Mol. Cell. 2019; 74:494–507. 30930054 10.1016/j.molcel.2019.02.034

[103] Li K. , Smagula C.S., Parsons W.J., Richardson J.A., Gonzalez M., Hagler H.K., Williams R.S. Subcellular partitioning of MRP RNA assessed by ultrastructural and biochemical analysis. J. Cell Biol. 1994; 124:871–882. 7510714 10.1083/jcb.124.6.871 PMC2119977

[104] Xue D. , Shi H., Smith J.D., Chen X., Noe D.A., Cedervall T., Yang D.D., Eynon E., Brash D.E., Kashgarian M. et al. A lupus-like syndrome develops in mice lacking the Ro 60-kDa protein, a major lupus autoantigen. Proc. Natl. Acad. Sci. U.S.A. 2003; 100:7503–7508. 12788971 10.1073/pnas.0832411100 PMC164616

[105] Rath S. , Sharma R., Gupta R., Ast T., Chan C., Durham T.J., Goodman R.P., Grabarek Z., Haas M.E., Hung W.H.W. et al. MitoCarta3.0: an updated mitochondrial proteome now with sub-organelle localization and pathway annotations. Nucleic Acids Res. 2021; 49:D1541–D1547. 33174596 10.1093/nar/gkaa1011 PMC7778944

[106] Chen X. , Taylor D.W., Fowler C.C., Galan J.E., Wang H.-W., Wolin S.L. An RNA degradation machine sculpted by Ro autoantigen and noncoding RNA. Cell. 2013; 153:166–177. 23540697 10.1016/j.cell.2013.02.037 PMC3646564

[107] Chen H.-W. , Rainey R.N., Balatoni C.E., Dawson D.W., Troke J.J., Wasiak S., Hong J.S., McBride H.M., Koehler C.M., Teitell M.A. et al. Mammalian polynucleotide phosphorylase is an intermembrane space RNase that maintains mitochondrial homeostasis. Mol. Cell Biol. 2006; 26:8475–8487. 16966381 10.1128/MCB.01002-06 PMC1636764

[108] Borowski L.S. , Dziembowski A., Hejnowicz M.S., Stepien P.P., Szczesny R.J. Human mitochondrial RNA decay mediated by PNPase-hSuv3 complex takes place in distinct foci. Nucleic Acids Res. 2013; 41:1223–1240. 23221631 10.1093/nar/gks1130 PMC3553951

[109] Engel K.L. , Lo H.-Y.G., Goering R., Li Y., Spitale R.C., Taliaferro J.M. Analysis of subcellular transcriptomes by RNA proximity labeling with Halo-seq. Nucleic Acids Res. 2021; 50:e24. 10.1093/nar/gkab1185 PMC8887463 34875090

[110] Herai R.H. , Negraes P.D., Muotri A.R. Evidence of nuclei-encoded spliceosome mediating splicing of mitochondrial RNA. Hum. Mol. Genet. 2017; 26:2472–2479. 28430982 10.1093/hmg/ddx142 PMC6075211

[111] Wang K. , Zhang S., Weber J., Baxter D., Galas D.J. Export of microRNAs and microRNA-protective protein by mammalian cells. Nucleic Acids Res. 2010; 38:7248–7259. 20615901 10.1093/nar/gkq601 PMC2978372

[112] Arroyo J.D. , Chevillet J.R., Kroh E.M., Ruf I.K., Pritchard C.C., Gibson D.F., Mitchell P.S., Bennett C.F., Pogosova-Agadjanyan E.L., Stirewalt D.L. et al. Argonaute2 complexes carry a population of circulating microRNAs independent of vesicles in human plasma. Proc. Natl. Acad. Sci. U.S.A. 2011; 108:5003–5008. 21383194 10.1073/pnas.1019055108 PMC3064324

[113] Turchinovich A. , Weiz L., Langheinz A., Burwinkel B. Characterization of extracellular circulating microRNA. Nucleic Acids Res. 2011; 39:7223–7233. 21609964 10.1093/nar/gkr254 PMC3167594

[114] Tosar J.P. , Gámbaro F., Darré L., Pantano S., Westhof E., Cayota A. Dimerization confers increased stability to nucleases in 5’ halves from glycine and glutamic acid tRNAs. Nucleic Acids Res. 2018; 46:9081–9093. 29893896 10.1093/nar/gky495 PMC6158491

[115] Théry C. , Witwer K.W., Aikawa E., Alcaraz M.J., Anderson J.D., Andriantsitohaina R., Antoniou A., Arab T., Archer F., Atkin-Smith G.K. et al. Minimal information for studies of extracellular vesicles 2018 (MISEV2018): a position statement of the International Society for Extracellular Vesicles and update of the MISEV2014 guidelines. J. Extracell. Vesicles. 2018; 7:1535750. 30637094 10.1080/20013078.2018.1535750 PMC6322352

[116] Mateescu B. , Kowal E.J.K., van Balkom B.W.M., Bartel S., Bhattacharyya S.N., Buzás E.I., Buck A.H., de Candia P., Chow F.W.N., Das S. et al. Obstacles and opportunities in the functional analysis of extracellular vesicle RNA - an ISEV position paper. J. Extracell. Vesicles. 2017; 6:1286095. 28326170 10.1080/20013078.2017.1286095 PMC5345583

[117] Driedonks T.A.P. , Mol S., de Bruin S., Peters A.-L., Zhang X., Lindenbergh M.F.S., Beuger B.M., van Stalborch A.-M.D., Spaan T., de Jong E.C. et al. Y-RNA subtype ratios in plasma extracellular vesicles are cell type- specific and are candidate biomarkers for inflammatory diseases. J. Extracell. Vesicles. 2020; 9:1764213. 32944168 10.1080/20013078.2020.1764213 PMC7448942

[118] Nechooshtan G. , Yunusov D., Chang K., Gingeras T.R. Processing by RNase 1 forms tRNA halves and distinct Y RNA fragments in the extracellular environment. Nucleic Acids Res. 2020; 48:8035–8049. 32609822 10.1093/nar/gkaa526 PMC7430647

[119] Tosar J.P. , Segovia M., Castellano M., Gámbaro F., Akiyama Y., Fagúndez P., Olivera Á., Costa B., Possi T., Hill M. et al. Fragmentation of extracellular ribosomes and tRNAs shapes the extracellular rnaome. Nucleic Acids Res. 2020; 48:12874–12888. 32785615 10.1093/nar/gkaa674 PMC7736827

[120] Sim S. , Wolin S.L. Bacterial Y RNAs: gates, tethers, and tRNA mimics. Microbiol. Spectr. 2018; 6:10.1128/microbiolspec.RWR-0023-2018. PMC6047535 30006996

[121] Cozen A.E. , Quartley E., Holmes A.D., Hrabeta-Robinson E., Phizicky E.M., Lowe T.M. ARM-seq: AlkB-facilitated RNA methylation sequencing reveals a complex landscape of modified tRNA fragments. Nat. Methods. 2015; 12:879–884. 26237225 10.1038/nmeth.3508 PMC4553111

[122] Zheng G. , Qin Y., Clark W.C., Dai Q., Yi C., He C., Lambowitz A.M., Pan T. Efficient and quantitative high-throughput tRNA sequencing. Nat. Methods. 2015; 12:835–837. 26214130 10.1038/nmeth.3478 PMC4624326

