# Supplementary material for: CoLoC-seq probes the global topology of organelle transcriptomes
Source: Nucleic Acids Res. 2022 Dec 20;51(3):e16. doi: 10.1093/nar/gkac1183 (PMC9943681; doi:10.1093/nar/gkac1183)
Supplement: gkac1183_Supplemental_Files [file gkac1183_supplemental_files.zip › Supplementary data_v04.pdf]

CoLoC-seq, a high-throughput approach to profile organelle transcriptomes

Damien Jeandard, Anna Smirnova, Akinyemi Mandela Fasemore, Léna Coudray, Nina Entelis, Konrad U. Förstner, Ivan Tarassov, Alexandre Smirnov

SUPPLEMENTARY DATA

Supplementary tables

Supplementary Table S1. DNA oligonucleotides used in this study

**Oligonucleotide ID**	**Sequence (5'-3')**	**Note**
A1 (*Homo sapiens COX1*) (Thermo Fisher Scientific, Waltham, USA, Cat # VA6-12665)	NA (25 oligo pair covering mtDNA positions 5923-7254)	bDNA smFISH probe for the *MT-CO1* mRNA
A3 (*Homo sapiens* RMRP) (Thermo Fisher Scientific, Waltham, USA, Cat # VA6-60000271)	NA (1 oligo pair covering gene positions 140-227)	bDNA smFISH probe for RMRP
A4 (*Homo sapiens* 5S) (Thermo Fisher Scientific, Waltham, USA, Cat # VA6-3173307)	NA (2 oligo pairs covering gene positions 2-118)	bDNA smFISH probe for 5S rRNA
A5 (*Homo sapiens* 5,8S) (Thermo Fisher Scientific, Waltham, USA, Cat # VA6-3170756)	NA (2 oligo pairs covering gene positions 2-139)	bDNA smFISH probe for 5.8S rRNA
A13 (*Homo sapiens* mitochondria) (Thermo Fisher Scientific, Waltham, USA, Cat # VA6-6000755)	NA (1 oligo pair covering mtDNA positions 1603-1655)	bDNA smFISH probe for mt-tRNA^{Val}
A15 (*Homo sapiens* RNY1) (Thermo Fisher Scientific, Waltham, USA, Cat # VA6-3173328-VCP)	NA	bDNA smFISH probe for Y1 RNA
A16 (*Homo sapiens* RNY3) (Thermo Fisher Scientific, Waltham, USA, Cat # VA6-3173329-VCP)	NA	bDNA smFISH probe for Y3 RNA
Anja0062	GTTGTTGGTACCATGAGGAAGATGCTCGCCG	Recloning PDHA1-mCherry into pcDNA5 FRT/TO, KpnI site
Anja0063	GTTGTTCTCGAGTTACTTGTACAGCTCGTCCATGCCG	Recloning PDHA1-mCherry into pcDNA5 FRT/TO, XhoI site
cKhOXC_rev	ACTTGAACCCTGGACC	Probe for cytosolic tRNA$^{Lys}_{TTT}$
Hm5ST7_R	AAAGCCTACAGCACCCGGTATTCCC	Probe for 5S rRNA
HmT7tRNA_Val_R	TGGGTCAGAGCGGTCAAGTTAAGTTGAAATCTCC	Probe for mitochondrial tRNA^{Val}
LysMtNeCyclrev	GGTCACTGTAAAGAGGTG	Probe for mitochondrial tRNA^{Lys}
oJD085	GGCCGCAAGTGCGTTCGAAG	Probe for 5.8S rRNA

oJD099	TGGAGCCCTGTAGGGGGCTCGAAC	Probe for the spike-in RNA
SAO00074	AAAATATGGAACGCTTCACGAATTTGC	Probe for U6 snRNA
SAO00133	CTCATGGTAGGGGTAAAAGG	Probe for the *MT-ND3* mRNA
SAO00331	CACTAGACCACCAGGGA	Probe for $tRNA^{Glu}_{CTC1}$
SAO00333	CTCACCACTATACTAACGAGGA	Probe for $tRNA^{Asp}_{GTC2}$
SAO00404	GAAGGAACAAAGAAATCTGTAACTGG TTGTGATCAATTAGTTGTAAAC	Probe for Y3 RNA
SAO00405	AAGACTAGTCAAGTGCAGTAGTGAG	Probe for Y1 RNA
TruSeq_Sense_primer	AATGATACGGCGACCACCGAGATCTAC ACNNNNNNNNACACTCTTTCCCTACAC GACGCTCTTCCGATCT	TruSeq sequencing primer (NNNNNNNN corresponds to an i5 barcode)
TruSeq_Antisense_primer	CAAGCAGAAGACGGCATACGAGATGT GACTGGAGTTCAGACGTGTGCTCTTCC GATCT	TruSeq sequencing primer

Supplementary Table S2. CoLoC-seq and Mock CoLoC-seq profiles of human mitochondria-associated RNAs and their kinetics parameters. "Gene ID" for compounded multicopy genes corresponds to one type entry for the entire group. The CoLoC-seq #1 and Mock CoLoC-seq #1 datasets are matched (i.e. performed on the same mitochondrial preparation); so are CoLoC-seq #2 and Mock CoLoC-seq #2. "Model" corresponds to either the regression Model 1 (complete, with P_0 and *k'* as parameters) or 2 (simple, with *k'* as the only parameter), as described in Materials and Methods. The Model 2 was used only in those few cases where Model 1 could not produce a converging fit. In those cases where the unconstrained Model 2 did not yield significant positive *k'* (meaning that there was no measurable degradation observed), P_0 was assigned the nominal value of [1.0000]. If, however, *k'* was significant but abnormally small (much smaller than the corresponding parameter for one of the poorest known RNase A substrates, see Supplementary Figure 7B), P_0 was assigned the nominal value of [0.5000].

Supplementary figures

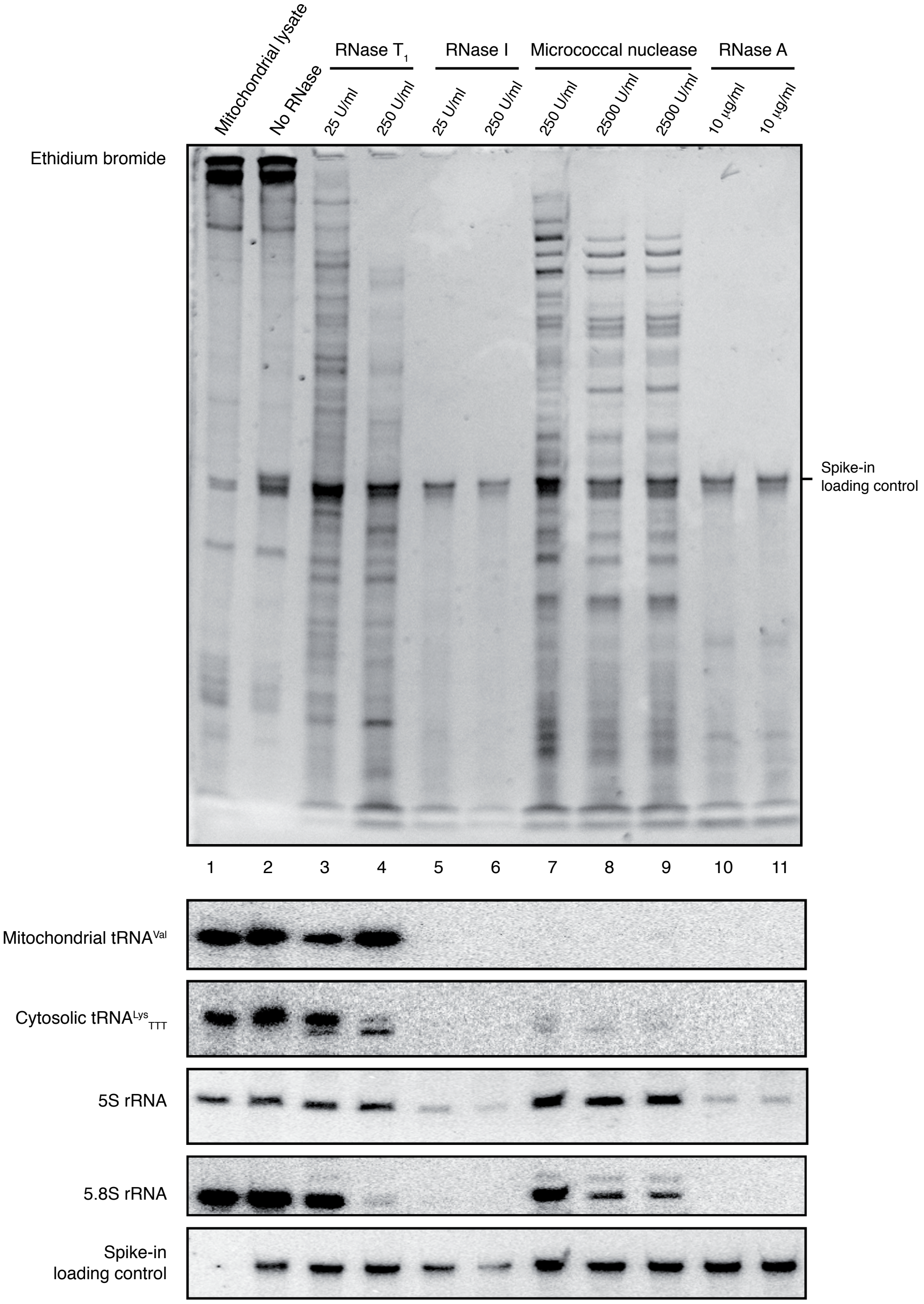

Supplementary Figure S1. Different RNases have unequal ability to degrade transcripts in mitochondrial lysates. Mitochondria of Flp-In T-REx 293 cells were lysed under native conditions and treated with various ribonucleases, as described in Materials and Methods. Isolated RNA was spiked-in and analysed by denaturing gel electrophoresis followed by ethidium bromide staining (*above*) and northern blotting with probes to selected abundant transcripts (*below*). Lane 1 shows RNA from an untreated mitochondrial lysate. Lanes 2-11 correspond to treated and spiked-in samples. For the Ca^{2+}-dependent micrococcal nuclease, the following buffers were used: 10 mM Tris-HCl, 0.6 M sorbitol, 10 mM $CaCl_2$, 1 mM PMSF, pH6.7 in lanes 7-8, and 10 mM Tris-HCl, 0.6 M sorbitol, 10 mM $CaCl_2$, 1 mM PMSF, pH7.5 in lane 9. Lanes 10-11 correspond to two different batches of RNase A. Note that 5S rRNA cannot be completely eliminated within the time frame of the experiment (10 min) even with the most active RNases I and A.

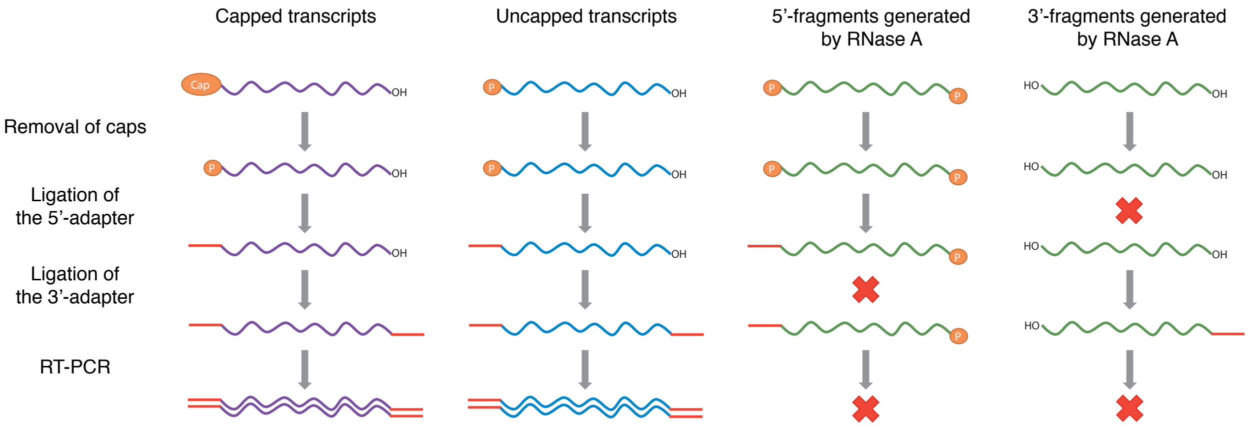

Supplementary Figure S2. CoLoC-seq library preparation strategy. The CoLoC-seq model relies on the quantification of intact transcripts. To distinguish between intact transcripts (most of which have a cap or a phosphate on the 5’-end and a hydroxyl on the 3’-terminus) and those cleaved at least once by RNase A (featuring 5’-hydroxyls and 3’-phosphates, one can, upon cap removal with RNA 5’-pyrophosphohydrolase, exploit differential adaptor ligation. The 5’-adaptor cannot be ligated to 5’-hydroxylated fragments, whereas the 3’-adaptor cannot be attached to a phosphate-blocked 3’-end. Thereby both 5’- and 3’-fragments generated by RNase A are effectively excluded from the final library; only cDNAs derived from intact transcripts get amplified and sequenced.

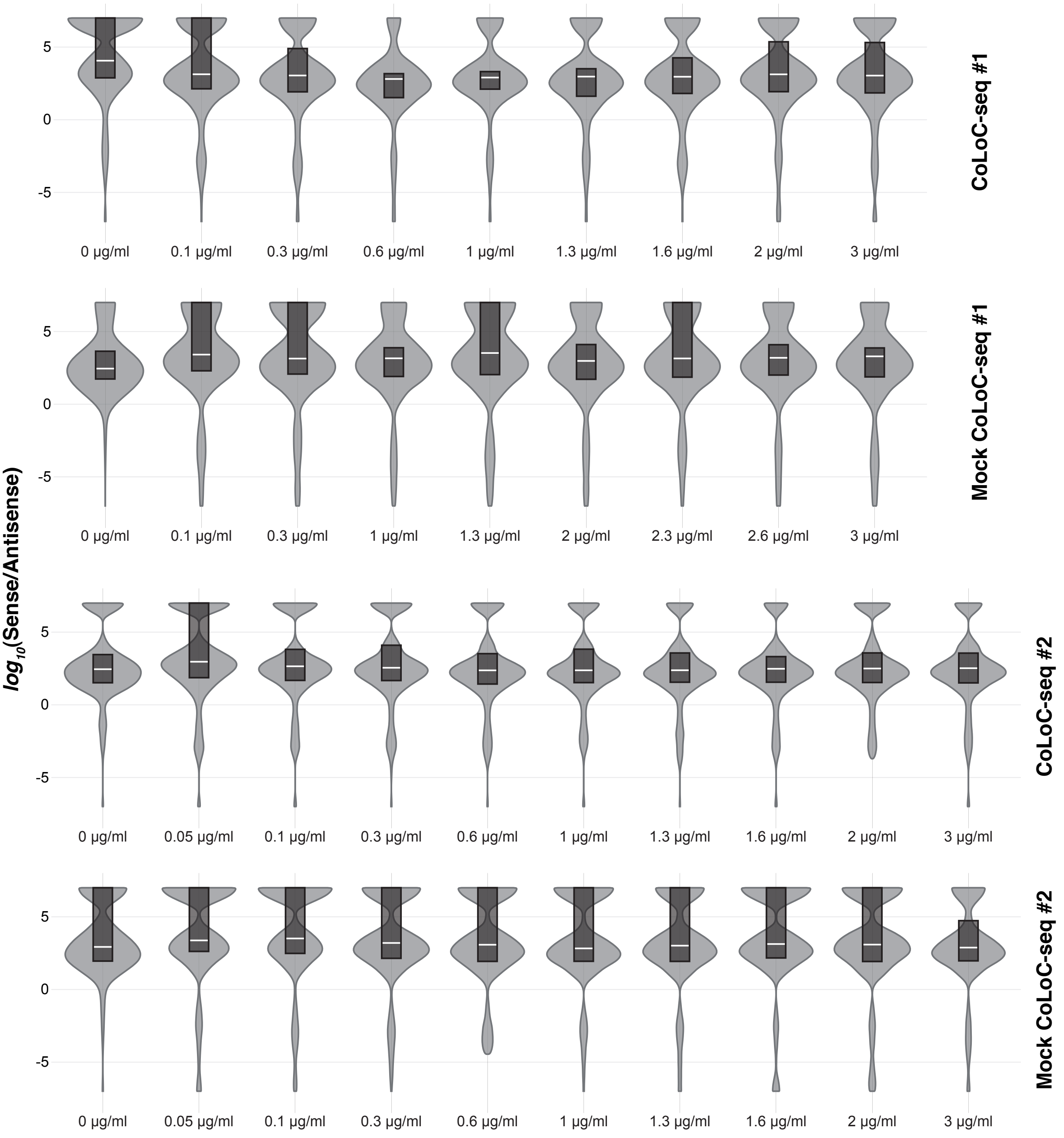

Supplementary Figure S3. Strandedness of CoLoC-seq libraries. For each expressed locus (covered with at least 50 reads on one of the strands), numbers of reads mapping in sense were divided by that of reads mapping in antisense. The box plot shows the median and the interquartile range. The Epanechnikov kernel density distributions are clamped to the maximum (0 reads in antisense) and the minimum (0 reads in sense). In all cases, the overwhelming majority of reads comes from the sense strand (log_{10}[*Sense/Antisense*] > 0).

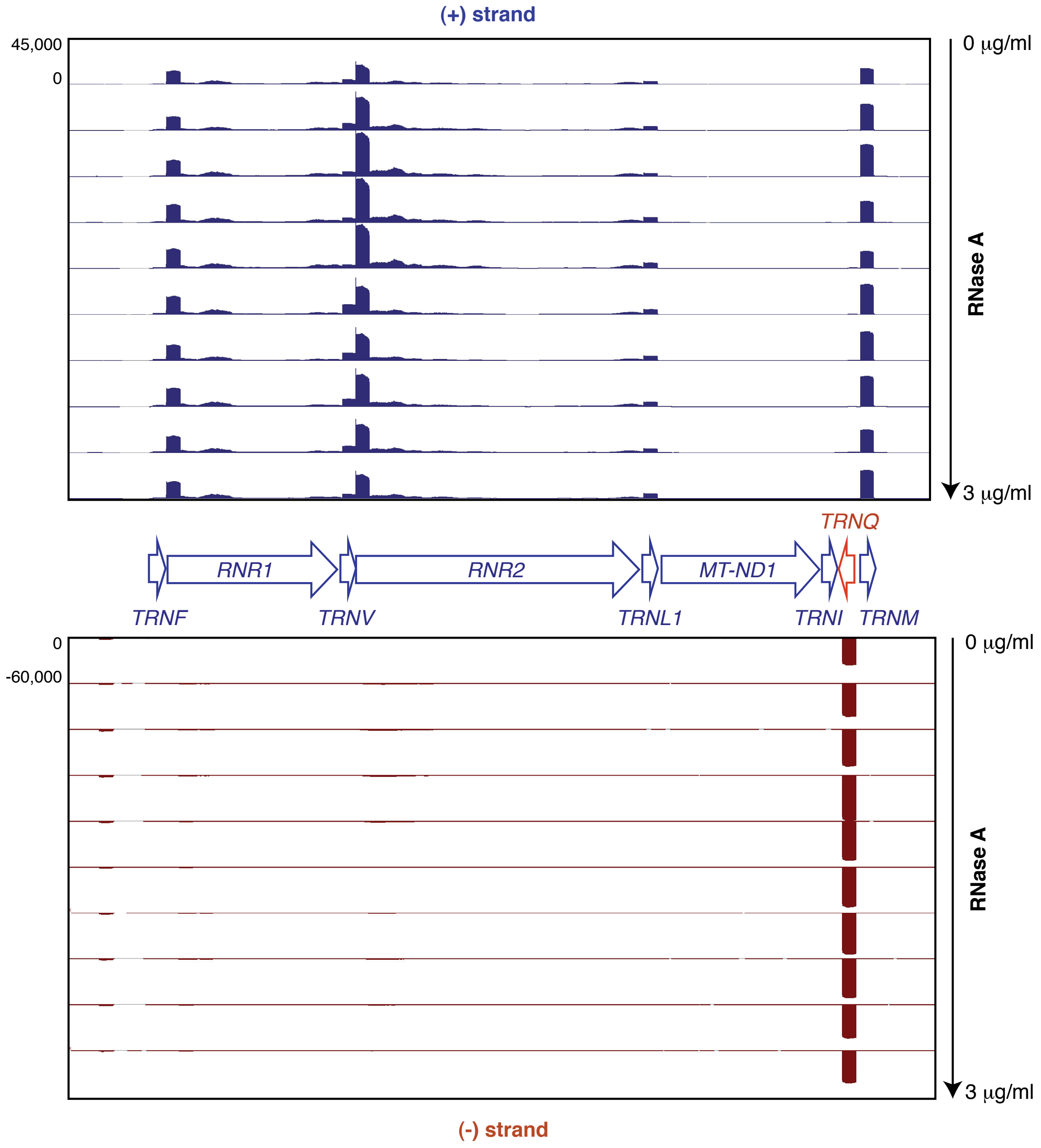

Supplementary Figure S4. CoLoC-seq reads cluster on the 5'-termini of transcripts in the sense orientation. Shown is the rRNA region of the mitochondrial genome profiled in a CoLoC-seq pipeline. The two ribosomal RNA genes (*RNR1* encoding 12S rRNA and *RNR2* specifying 16S rRNA) are abundantly expressed but the reads accumulate primarily on the 5'-end, where the first adaptor was ligated (see Supplementary Figure 2). Note that the only (-) strand gene in this locus is *TRNQ*, which is correctly captured by the orientation of mapped reads, indicating the preservation of the strand information in CoLoC-seq libraries.

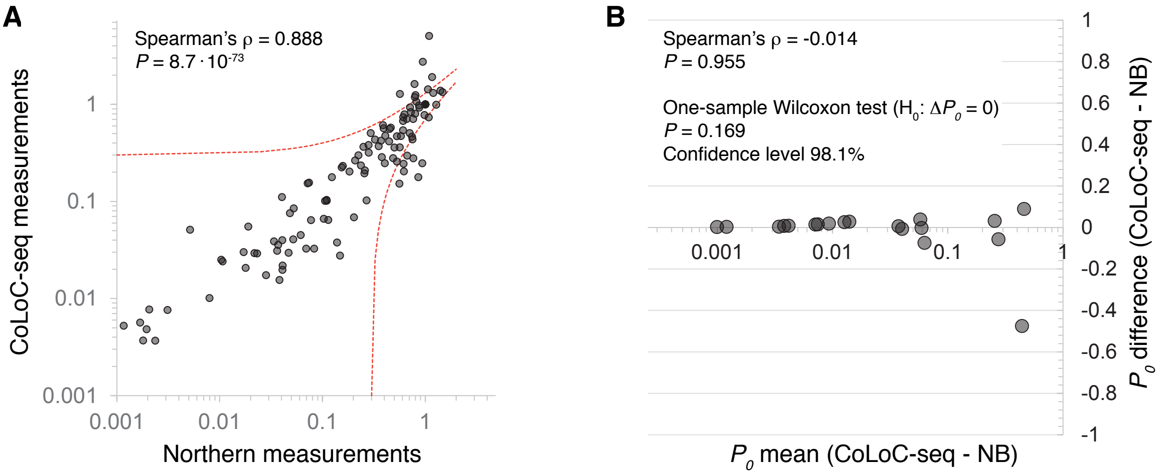

Supplementary Figure S5. CoLoC-seq measurements of RNA depletion dynamics agree with those by a conventional approach. (A) CoLoC-seq and northern blotting provide concordant measurements of RNA levels in individual fractions. Read counts and northern blot signals from 7 different RNAs were measured in 2 independent CoLoC-seq and 2 Mock CoLoC-seq experiments (*n* = 212 individual measurements), normalised by the corresponding level of the spike-in RNA, and plotted against each other. The red lines delimit a zone with a deviation of <0.3 from the diagonal. **(B)** Blant-Altman plot showing the concordance in P_0 determinations between CoLoC-seq and northern blotting. P_0 values (*n* = 19) were obtained by fitting the Model 1 (see Materials and Methods) into the depletion profiles of 7 different transcripts from 2 independent CoLoC-seq and 2 Mock CoLoC-seq experiments, as measured by deep sequencing and by northern blotting (NB).

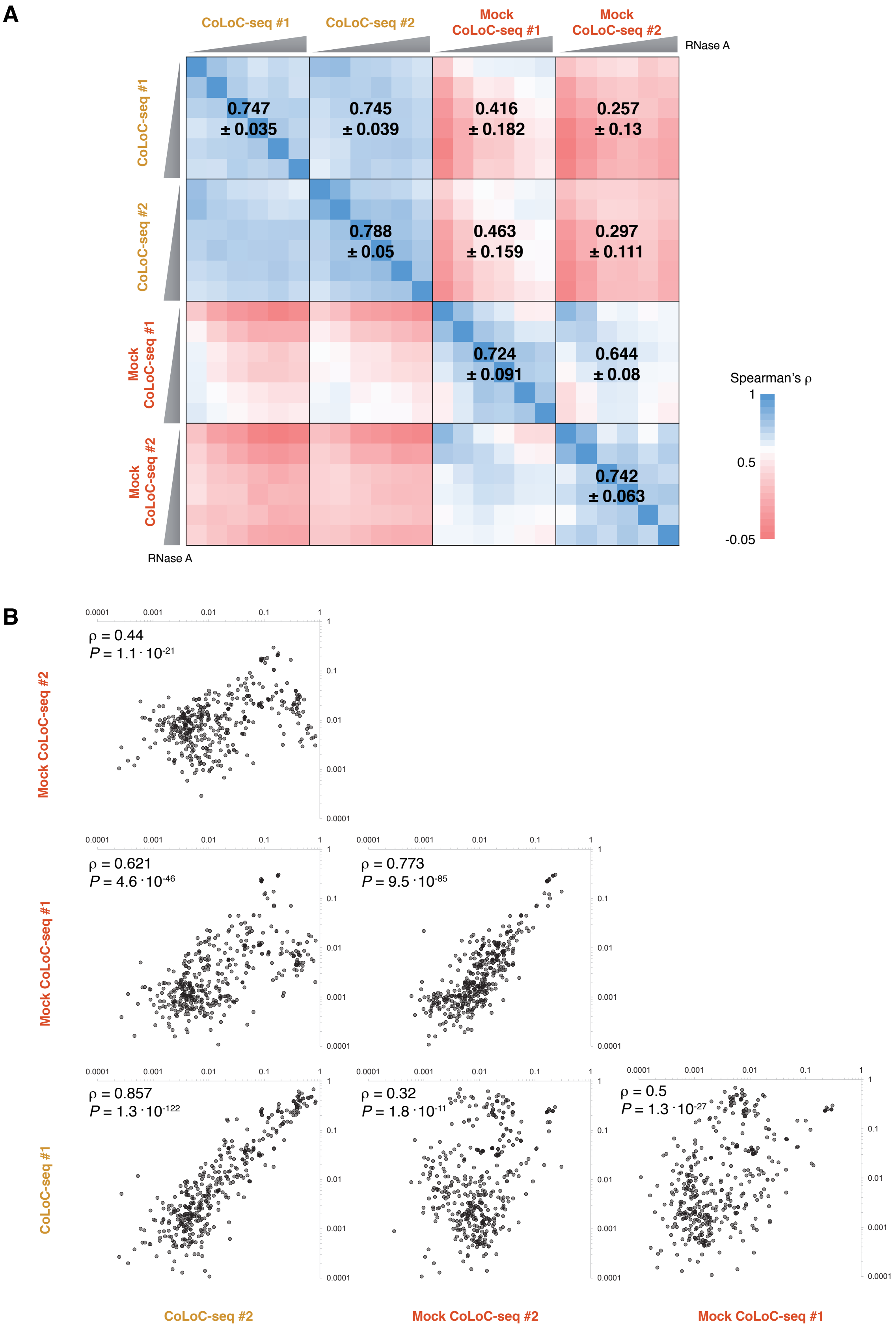

Supplementary Figure S6. Intra-method reproducibility of CoLoC-seq. (A) Remaining RNA proportions in RNase A-treated samples from 2 independent CoLoC-seq and 2 independent Mock CoLoC-seq replicates were compared with each other in all possible combinations. Replicates labelled #1 are paired with each other, so are

those labelled #2. Small squares correspond to individual sample comparisons (only the samples with the same RNase concentrations across all conditions are shown: 0.1, 0.3, 1, 1.3, 2, and 3 µg/ml). In each large square corresponding to a group of all cross-sample comparisons from two specific experiments, the mean (± SD) Spearman's correlation coefficient (excluding redundant comparisons) is provided. The CoLoC-seq samples are similar to each other, so are the Mock CoLoC-seq samples. By contrast, the CoLoC-seq samples differ markedly from the Mock CoLoC-seq ones. **(B)** P_0 values independently estimated by fitting the Model 1 into the data from two different CoLoC-seq and two different Mock CoLoC-seq replicates were compared with each other. The two CoLoC-seq replicates largely agree with each other, and the two Mock CoLoC-seq replicates similarly give concordant P_0 estimates. By contrast, the CoLoC-seq P_0 estimates are only marginally similar to the Mock CoLoC-seq ones, indicating that even though the majority of RNAs are degraded similarly in both experiments (with low P_0 values), the behaviour of a significant group of transcripts (i.e. mitochondria-resident RNAs) is different between the two setups.

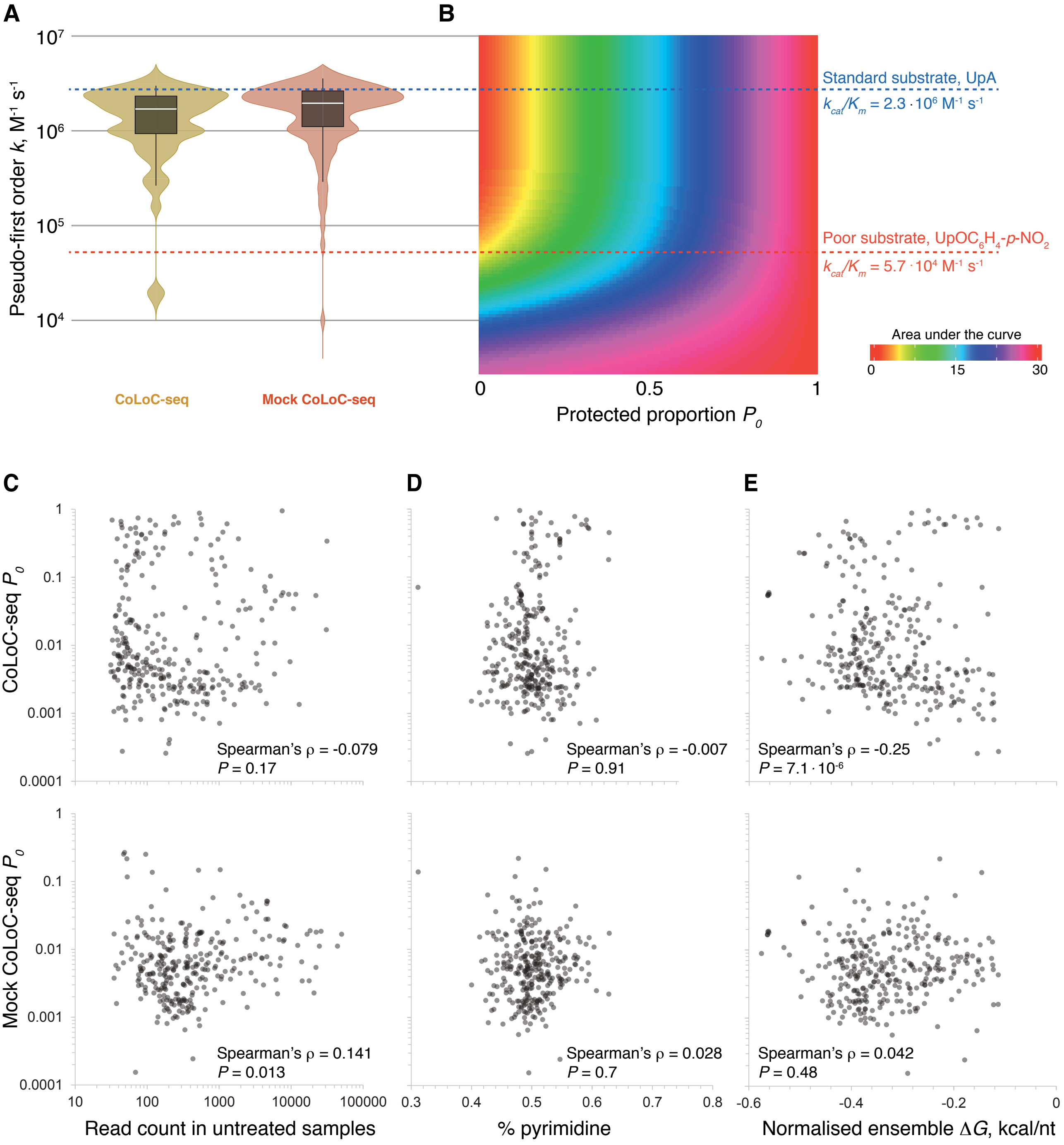

Supplementary Figure S7. Kinetics parameters of RNase A-mediated RNA digestion observed in CoLoC-seq experiments are biochemically relevant and enable unbiased estimation of the size of protected pools. **(A)** Distribution of digestion rate constants derived from CoLoC-seq and Mock CoLoC-seq measurements (Supplementary Table S2). The box plot shows the median, the interquartile range, the 5%-95% percentile range, and the Epanechnikov kernel density distribution (n = 289 for CoLoC-seq, n = 319 for Mock CoLoC-seq; non-significant rates of fully protected mtDNA-encoded transcripts and abnormally high apparent rates of too fast-digested contaminants were excluded from the analysis). **(B)** Model 1 was used to simulate RNA depletion curves resulting from a wide variety of k *vs* P_0 combinations (see Materials and Methods). The corresponding areas under the curve were colour-coded and plotted. The observed isotherms indicate similarly looking profiles that can be obtained from different combinations of these parameters. In the region of the plot where the isotherms are vertical ($k > 10^5$ M^{-1} s^{-1}), P_0 is not entangled with k anymore and can be estimated with precision. For reference, the experimentally determined k_{cat}/K_m values (which for kinetically perfect enzymes approximate the reaction k_{on})

measured for two model RNase A substrates under similar reaction conditions (69, 83) are indicated. **(C-E)** Absence of appreciable bias in P_0 determination by CoLoC-seq and Mock CoLoC-seq with respect to the apparent abundance (C), pyrimidine content (D), and the overall foldedness (E) of transcripts (*n* = 308 clearly defined transcripts with annotated boundaries). Abundance was measured as the average number of reads in untreated samples. Foldedness was calculated as transcript length-normalised ensemble folding free energy, as predicted by RNAfold (75). For spliced transcripts, isoform 1 was chosen as representative.

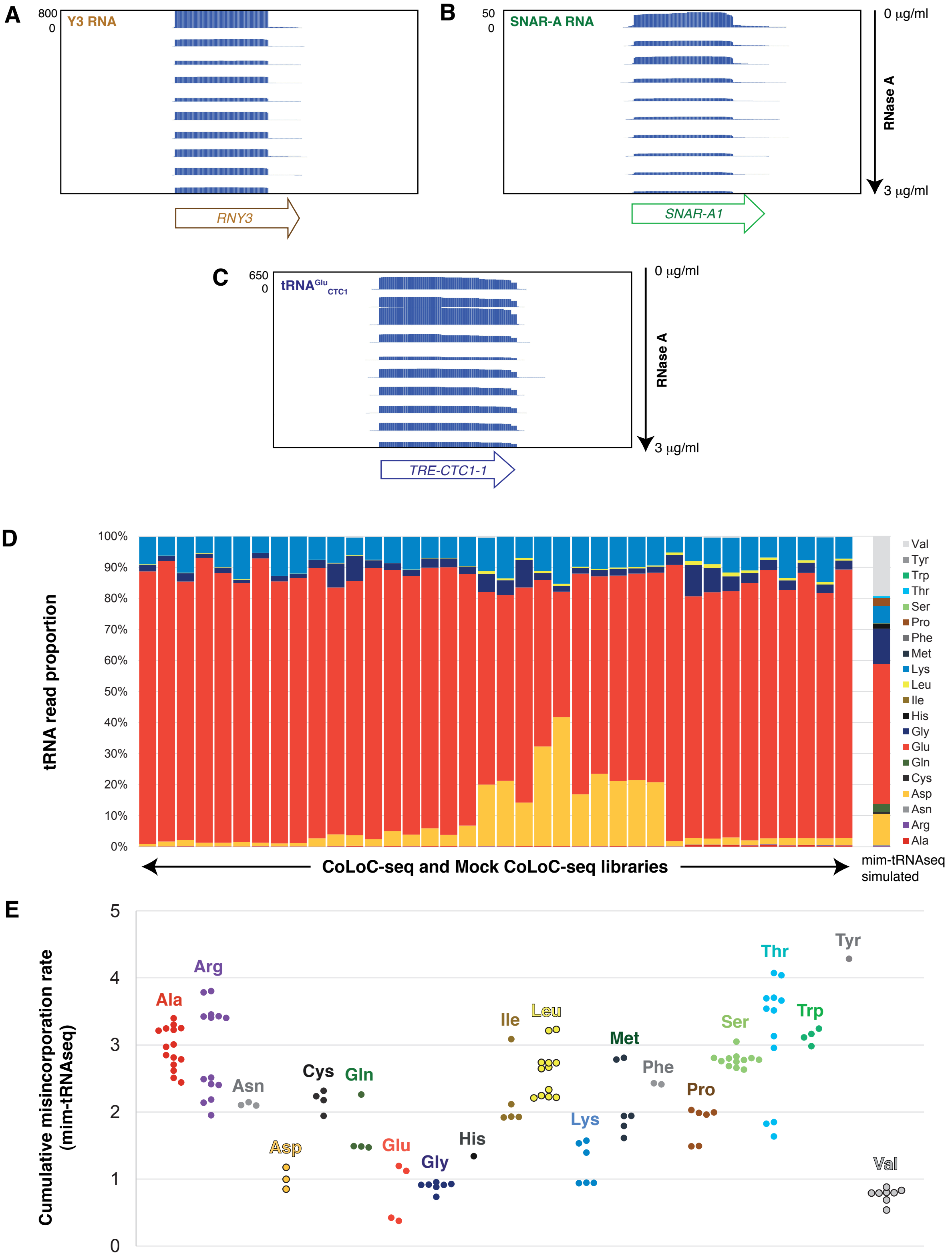

Supplementary Figure S8. nDNA-encoded mitochondria-resident transcripts. (A-C) Examples of CoLoC-seq read coverages across the genomic loci encoding Y3 RNA (A), SNAR-A RNA (B), and $tRNA^{Glu}_{CTC1}$ (C), suggesting that they are mostly present as full-length transcripts. **(D)** Limited tRNA diversity (by amino acid

specificity) of CoLoC-seq and Mock CoLoC-seq libraries. Shown are the sequenced tRNA compositions of the 38 CoLoC-seq and Mock CoLoC-seq libraries presented in this study. On the right, for comparison, a simulated total tRNA library is shown, as if it were produced by a standard reverse transcriptase, which drops off at modified positions with probabilities proportional to the modification level (based on true tRNA abundances and TGIRT misincorporation rates measured by mim-tRNAseq in human iPSC cells (96)). Note that the resulting distorted distribution singles out the same tRNAs as apparently the most abundant: Glu, Gly, Asp, Lys, but also Val. $tRNA^{Val}$ are much less frequently sequenced in CoLoC-seq and Mock CoLoC-seq libraries than expected from this simulation, likely due to a hard stop caused by a dihydrouridine in position 20e (97), which cannot be measured by mim-tRNAseq. **(E)** Cumulative TGIRT misincorporation rates reported for human iPSC cells by mim-tRNAseq for individual tRNA isodecoders (96). Misincorporation rates per modified position were summed up for each tRNA to provide a measure of its overall modification level.

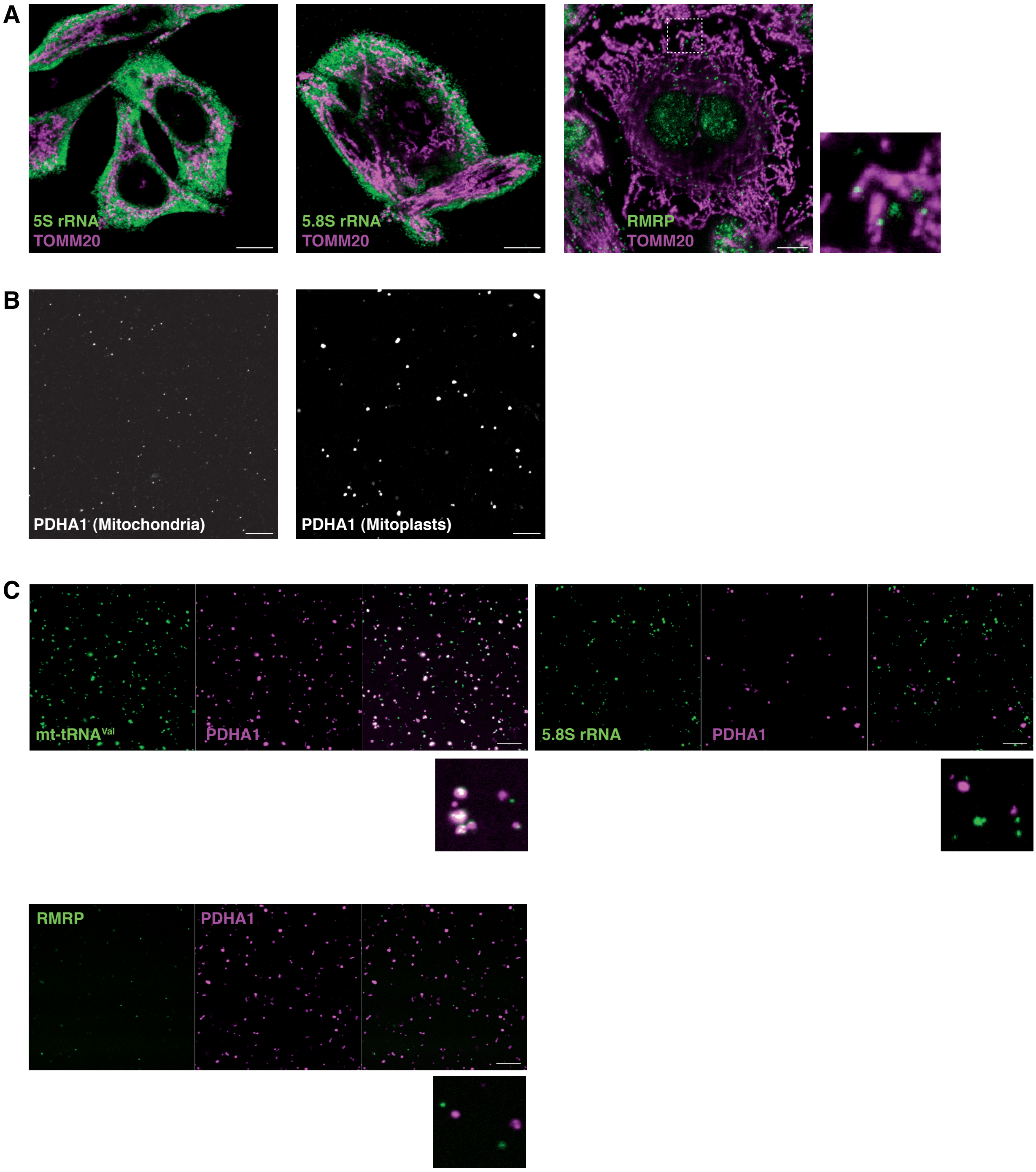

Supplementary Figure S9. smFISH analysis of select mitochondria-associated transcripts. (A) RMRP, 5S and 5.8S rRNAs were detected in HepG2 cells by branched DNA smFISH and visualised under a confocal microscope (*green*). TOMM20, an outer mitochondrial membrane protein, was visualised by immunofluorescence (*magenta*). Size bar is 10 µm. **(B)** Digitonin-mediated disruption of the outer mitochondrial membrane (mitoplasting) increases the size of isolated mitochondria, facilitating their observation and colocalization studies. Mitochondria and mitoplasts were isolated from HEK293 cells expressing the mitochondrial matrix-localised PDHA1-mCherry fusion. **(C)** Mitoplasts of HEK293 cells expressing PDHA1-mCherry were prepared from crude RNase A-treated mitochondria by digitonin-mediated disruption of the outer membrane and subjected to smFISH. Size bar is 10 µm. Zoom-in views (10×10 µm) are provided for select cases of colocalization (*white*) or of absence thereof.

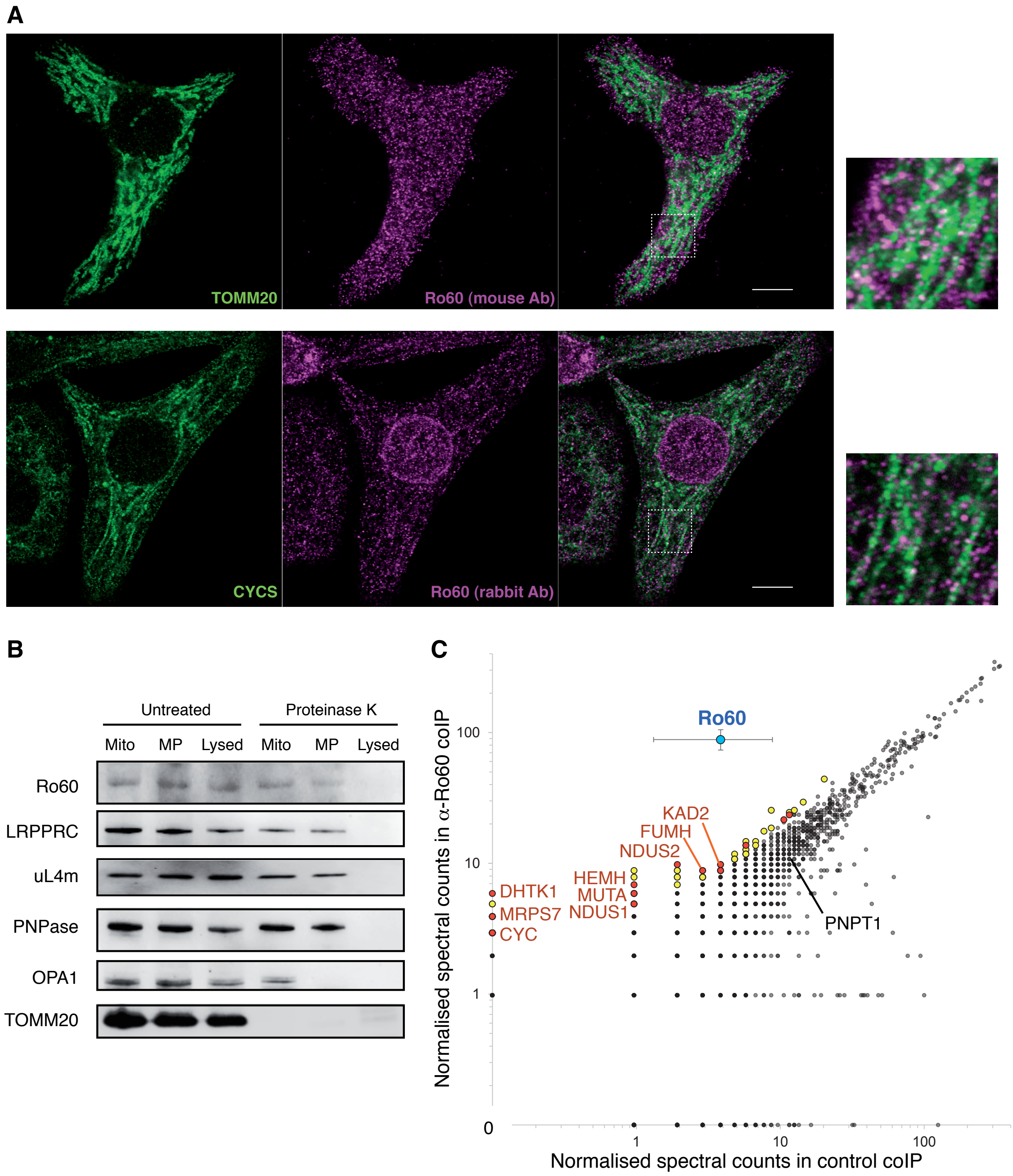

Supplementary Figure S10. Ro60 is likely present in the mitochondrial matrix. (A) Subcellular distribution of Ro60 was analysed in HepG2 cells by immunofluorescence with two different antibodies (rabbit polyclonal, Sigma-Aldrich, Cat # HPA002835, or mouse monoclonal, Santa Cruz Biotechnology, Inc, Cat # sc-100844), counterstained for mitochondrial markers (TOMM20, cytochrome c). Size bar is 10 µm. Zoom-in views (10×10 µm) are provided for select cases of colocalization (white) or of absence thereof. **(B)** Submitochondrial fractionation of mitochondria-associated proteins. Intact mitochondria, mitoplasts (MP), or lysed mitochondria, obtained from HEK293 cells as in Fig. 5A, were treated with proteinase K or left untreated. Surviving proteins were analysed by western blotting. LRPPRC and uL4m are matrix proteins. PNPase was reported to localise in both the matrix and the IMS. OPA1 is an inner membrane protein exposed into the IMS. TOMM20 is an outer membrane protein facing the cytosol. Ro60 was detected with a Human Protein Atlas rabbit polyclonal antibody (Sigma-Aldrich, HPA002835). **(C)** The mouse monoclonal α-Ro60 antibody (Santa Cruz Biotechnology, Inc, Cat # sc-100844) specifically immunoprecipitates Ro60 from total HEK293 cell lysates. Select mitochondrial proteins significantly co-enriched with Ro60 are labelled. PNPase is not enriched. Bars are Poissonian 95% CIs.
